# Marigold Metabolites: Diversity and Separation Methods of *Calendula* Genus Phytochemicals from 1891 to 2022

**DOI:** 10.3390/molecules27238626

**Published:** 2022-12-06

**Authors:** Daniil N. Olennikov, Nina I. Kashchenko

**Affiliations:** Laboratory of Biomedical Research, Institute of General and Experimental Biology, Siberian Division, Russian Academy of Science, 670047 Ulan-Ude, Russia

**Keywords:** *Calendula*, marigold, metabolites, separation methods, chromatography

## Abstract

Marigold (*Calendula*), an important asteraceous genus, has a history of many centuries of therapeutic use in traditional and officinal medicines all over the world. The scientific study of *Calendula* metabolites was initiated at the end of the 18th century and has been successfully performed for more than a century. The result is an investigation of five species (i.e., *C. officinalis*, *C. arvensis*, *C. suffruticosa*, *C. stellata*, and *C. tripterocarpa*) and the discovery of 656 metabolites (i.e., mono-, sesqui-, di-, and triterpenes, phenols, coumarins, hydroxycinnamates, flavonoids, fatty acids, carbohydrates, etc.), which are discussed in this review. The identified compounds were analyzed by various separation techniques as gas chromatography and liquid chromatography which are summarized here. Thus, the genus *Calendula* is still a high-demand plant-based medicine and a valuable bioactive agent, and research on it will continue for a long time.

## 1. Introduction

**Figure 1:**
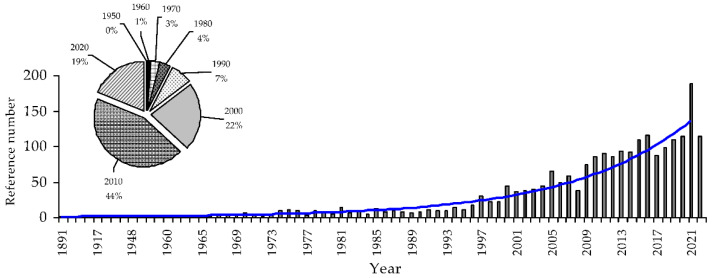
Distribution of studies on plant species from the *Calendula* genus by year (1891–2022) and an exponential ‘curve of interest’ (blue line). The X-axis is the year, and the Y-axis is the number of publications. The inset shows the impact of each decade on the total publication value.

*Calendula* (marigold; *Caléndula* L.) is a genus of herbaceous plants from the Asteraceae family, whose members are widely used for medicinal and decorative purposes. Genus *Calendula* includes 12 species of which *Calendula officinalis* L. is the most famous plant and the oldest medical remedy [1]. To date, experimental science has accumulated a considerable amount of scientific information about this genus; therefore, we performed a scientometric study of the available information. There are more than 2200 articles related to the study of the *Calendula* species for the period of 1891–2022 (Figure 1).

Statistical studies indicate an exponential growth in scientific interest in *Calendula*; the value of the determination coefficient (r^2^) for the ‘curve of interest’ (Y = 0.6718·e^0.0726·X^) is 0.9435, which indicates the reliability of these statements. Thus far, the greatest scientific impact on the total number of studies on *Calendula* was made during 2010–2019 (44% of publications); however, because during 2020–2022, approximately 19% of studies on this topic were completed, the picture may change in the near future. Among the scientific areas in which *Calendula* research is performed, the agricultural and biological (approximately 38% of publications), medical (approximately 28%), and pharmacology/toxicology sciences (approximately 25%) occupy a predominant position (Table S1). The largest number of works published by authors are from India (208), USA (200), Iran (189), Brazil (158), and Italy (148), and the authors with the largest number of articles are Kasprzyl Z. (35), Janiszowska W. (33), Szakiel A. (24), and Bransard G. (10). The top 10 most-cited articles with more than 100 citations include studies on chemical composition (triterpenoids, lipids), biological activity (anti-inflammatory, antioxidant, hypoglycemic), as well as clinical trials and allergic properties [2,3,4,5,6,7,8,9,10,11] (Table S2).

As expected, this level of scientific interest has led to the fact that review papers on various *Calendula* aspects are published in the scientific literature with varying frequency. In total, twelve reviews have been published from 2006 to 2022 (Table 1). All identified review articles had an important goal of generalizing data on the pharmacological activity of *Calendula* extracts to the detriment of information on the chemical composition. As a result, the total number of compounds mentioned in these works was 0–155. The work that cites the largest number of compounds (155) was published in 2009; therefore, this information needs to be updated. None of the reviews summarized data on the methods of analysis and/or separation of *Calendula* metabolites, which is a very important aspect of practical research of plant samples. Therefore, the aim of this work is to summarize the scientific information about the *Calendula* genus regarding the metabolite’s diversity as well as methods of analysis and separation.

## 2. Review Strategy

The resources of international databases (e.g., Scopus, Web of Science, PubMed, and Google Scholar) were used, and only original papers written in English and published in journals prior to October 2022 were considered. The search keywords used included plant names (e.g., “Calendula”, “Calendula officinalis”, etc.) and metabolite names. Metabolites with tentative structure (e.g., “quercetin-*O*-desoxyhexosyl-*O*-hexoside”, etc.) were excluded from the study. The structures of well-known metabolites (e.g., monoterpenes, sesquiterpenes, fatty acids, amino acids, etc.) are not discussed in this paper.

## 3. Chemodiversity of *Calendula* Genus

Some of the earliest chemical studies of the *Calendula* genus are the reports of F.A. Wirth (1891) [24], A. Kirchner (1892) [25], and A. Hilger (1894) [26] on the coloring pigments of *C. officinalis* flowers, which indicated the presence of phytosterols and some esters. Later, H. Kylin (1926) determined that the color of marigold flowers was primarily due to the carotenoid pigment calendulin, which differs from carotene; in 1932, L. Zechmeister and L. von Cholnoky characterized calendulin as a mixture of lycopene and violaxanthin [27]. Research on *C. officinalis* carotenoids was continued only in 1951 [28], after which investigations of the metabolites of this species and the genus became regular and have continued to this day for more than 70 years.

The chemical studies of *Calendula* genus metabolites include five species: *C. officinalis* or pot marigold (common marigold) is the most famous and widely distributed medicinal plant; *C. arvensis* or field marigold and *C. suffruticosa* or bush marigold are native to Central and Southern Europe; *C. stellata* or star marigold is grown in Northwestern Africa, Malta, and Sicily; and small tripterous marigold *C. tripterocarpum* occurs in Spain, Iran, and Africa. During 1892–2022, more than 650 compounds (**1**–**656**) have been identified for the genus *Calendula*, including monoterpenes (**1**–**44**), sesquiterpenes (**45**–**173**) and sesquiterpene glycosides (**174**–**207**), diterpenes (**208**, **209**), triterpenes (**210**–**342**), carotenoids (**343**–**437**), phenols (**438**–**443**), benzoic acid derivatives (**444**–**456**), hydroxycinnamates (**457**–**478**), coumarins (**479**–**488**), flavonols (**489**–**516**), anthocyanins (**517**–**524**), alkanes (**525**–**550**), aliphatic alcohols (**551**–**559**), aliphatic aldehydes and ketones (**560**–**565**), fatty acids and esters (**566**–**602**), chromanols (**603**–**613**), organic acids (**614**–**616**), carbohydrates (**617**–**630**), amino acids (**631**–**646**), and other groups (**647**–**656**) (Table 2). In addition, several polysaccharides have been isolated and characterized. Among the species mentioned, the most studied is *C. officinalis* for which 529 compounds are known, followed by *C. arvensis* (187 comp.), *C. suffruticosa* (68 comp.), *C. stellata* (27 comp.), and *C. tripterocarpa* (5 comp.). In terms of the organ-specific distribution of known metabolites of *C. officinalis*, the flowers are the best-studied part and are known to contain 403 compounds, while the leaves, roots, and seeds are known to contain 138 compounds. Studies on other species have been performed mainly on samples of the aerial part.

### 3.1. Monoterpenes

Monoterpenes **1**–**44** were found in the essential oils of *C. officinalis*, *C. arvensis*, and *C. stellata* herb, flowers, and leaves [16,29,30,31,32,33,34]. The typical compounds of the *Calendula* genus are linalool (**15**), limonene (**17**), β-myrcene (**21**), α/β-pinene (**27**/**29**), sabinene (**33**), γ-terpinene (**40**), terpinene-4-ol (**42**), α-terpinolene (**43**), and α-tujene (**44**) because these are routinely identified in essential oil samples using gas chromatography–mass spectrometry (GC-MS). These compounds are likely responsible for the characteristic odor of marigold flowers, although this has not been confirmed by olfactory analysis.

### 3.2. Sesquiterpenes

**Figure 2:**
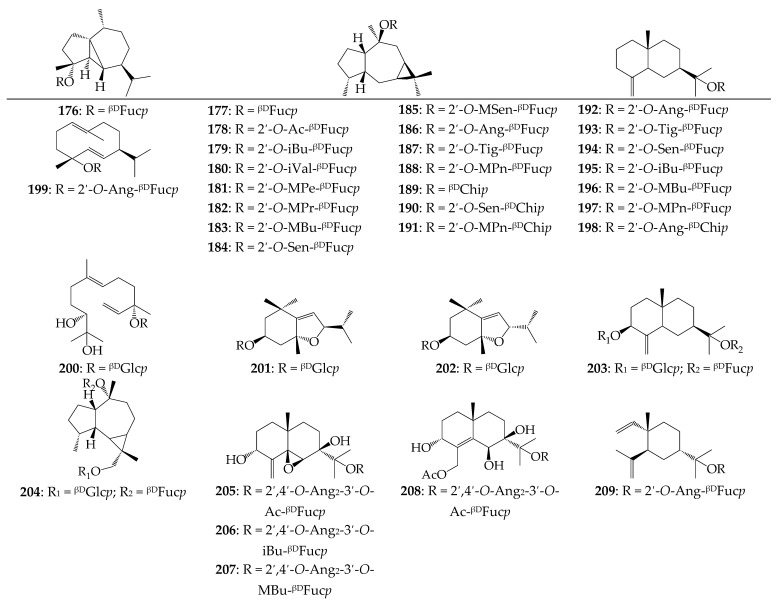
Sesquiterpenes **176**–**209**. Ac—acetyl; Ang—angeloyl; But—butyl; dCrt—dicrotaloyl; ^βD^Chi*p*—β-D-chinovopyranose; ^βD^Fuc*p*—β-D-fucopyranose; iBu—isobutyryl; iVal—isovaleroyl; ^βD^Glc*p*—β-D-glucopyranose; MBu—methylbutenoyl; MPe—methylpentenoyl; MPn—3-methyl-2-pentenoyl; MPr—methylpropanoyl; MSen—4-methylsenecioyl; Sen—senecioyl; Tig—tigloyl.

A total of 163 compounds of sesquiterpene nature were detected or isolated from four calendulas, i.e., 129 non-glycosidic compounds (**45**–**173**) and 34 glycosides (**174**–**207**) (Figure 2).

All non-glycosides were detected in the essential oils of *C. arvensis*, *C. officinalis*, and *C. suffruticosa* [30,31,37]. Structurally, derivatives of cadinane, carotane, caryophyllane, cubebane, eromophyllane, eudesmane, muurolane, and selinane dominated in all samples studied.

The sesquiterpene glycosides of the *Calendula* genus (a rare group of natural terpenoids) have attracted much greater interest. The first compound, arvoside A (**174**), isolated from *C. arvensis*, is a very rare 4-*epi*-cubebol glycoside [44]. Later, viridiflorol derivatives (**175**–**189**) were found in *C. arvensis* (as *C. persica*) and *C. officinalis*. This is the largest group of sesquiterpene glycosides in which hydroxyl can be substituted by fucose or chinovose acylated by acetic [44], isobutyric [45], isovaleric [44], methylpentenoic [44,46], methylpropanoic [47,48], methylbutenoic [46,47], senecic [45,46], 4-methylsenecic [46], angelic [45], and tiglic acids [45]. Similar to viridiflorol fucosides and chinovosides of β-eudesmol, **190**–**196** were identified in *C. arvensis* [46] and *C. officinalis* [45]. Rare angeloyl fucosides of 4α-hydroxygermacra-1(10)*E*,5*E*-diene (**197**) [46], α-elemol (**207**) [45], and 3α,7β-dihydroxy-5β,6β-epoxyeudesm-4(15)-ene (**203**–**208**) [48], as well as megastigmane glucosides officinoside A (**199**) and B (**200**) [50], icariside C_3_ (**198**) [9], and glucosyl fucosides officinoside C (**201**) and D (**202**) [50] showed the unique sesquiterpene profile of *Calendula* plants.

### 3.3. Diterpenes

Two diterpenes, neophytadiene (**176**) and phytol (**177**), were identified in the essential oils of *C. arvensis*, *C. officinalis*, and *C. suffruticosa* [40,41].

### 3.4. Triterpenes

**Figure 3:**
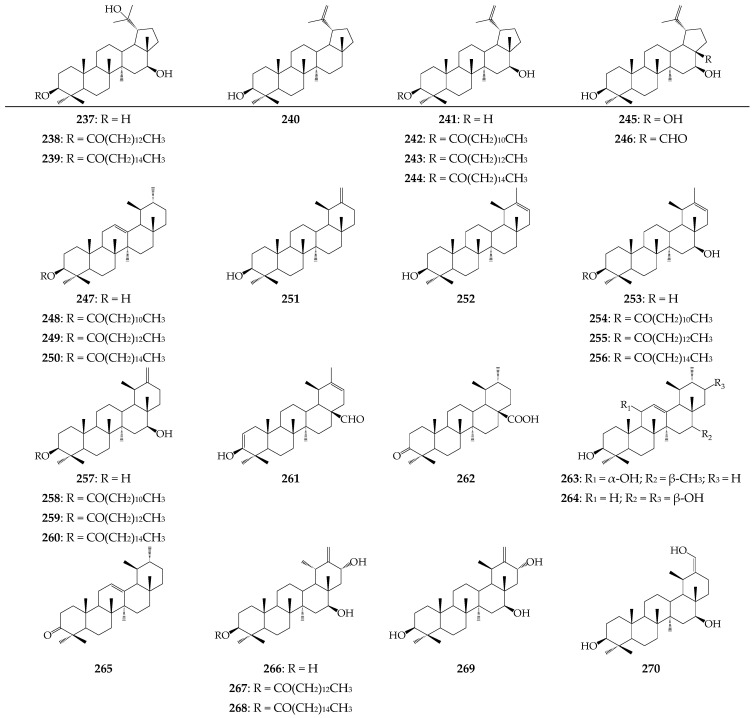
Lupane derivatives **237**–**246** and ursane derivatives **247**–**270**.

**Figure 4:**
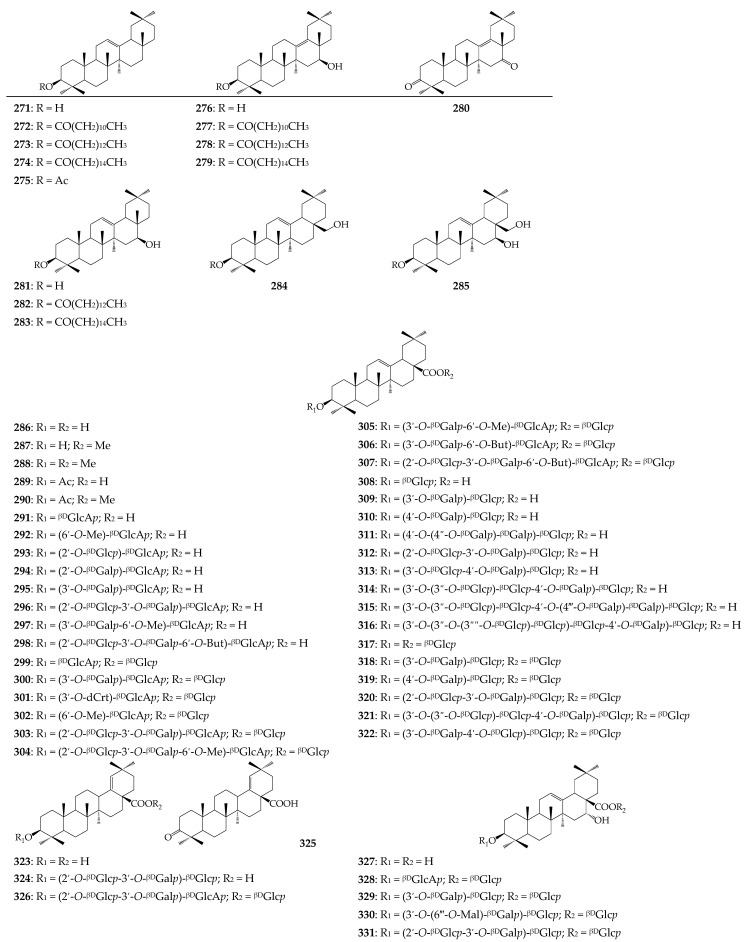

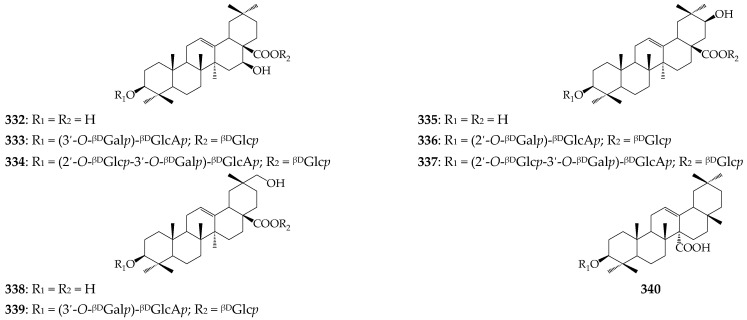
Oleanane derivatives **271**–**340**. Ac—acetyl; ^βD^Gal*p*—β-D-galactopyranose; ^βD^Glc*p*—β-D-glucopyranose; ^βD^GlcA*p*—β-D-glucuronopyranose; Mal—malonyl; Me—methyl.

Triterpenes of the genus *Calendula* are present in plants both in the free state and as esters with fatty acids (lauric, myristic, palmitic) or alcohols (methanol, *n*-butanol), as well as in the glycosidic form. Isolated and characterized compounds were derived from eleven parent structures, including stigmastane (**211**–**220**), ergostane (**221**–**224**), cholestane (**225**–**229**), lanostane (**230**, **231**), dammarane (**232**), cycloartane (**233**, **234**), fridelane (**235**, **236**), lupane (**237**–**246**; Figure 3), ursane (**247**–**270**; Figure 3), oleanane (**271**–**340**; Figure 4) and tirucallane (**341**, **342**). The only aliphatic triterpene squalene (**210**) was found in *C. suffruticosa* [42]. Stigmastanes, ergostanes, cholestanes, and lanostanes represent sterol derivatives of the *Calendula* genus that are most abundant in *C. officinalis* [51,58,59]. Cycloartanes, fridelanes, lupanes, and ursanes are non-glycosidic compounds that exist in the form of alcohols, aldehydes, and ketones. Selected lupanes (lupane-3β,16β,20-triol, calenduladiol) and ursanes (α-amyrin, faradiol, arnidiol, arnitriol) are esterified by lauric, myristic, and palmitic acids [53,56,60].

In the oleanane group, oleanolic acid (**286**) and derivatives (**287**–**322**) have shown the largest diversity. The structural features of oleanolic acid glycosides that distinguish *Calendula* from other Compositae species are the ability to form mono- and oligoglycosides with one and/or two points of attachment of carbohydrate fragments at the C-3 and C-28 positions. Two types of glycosides have been identified in *Calendula* plants, i.e., acidic and neutral. Acidic glycosides contain a glucuronic acid fragment at C-3, which can be linked to glucose and galactose at C-2′, galactose at C-3′, and/or esterified at C-6′ with methanol or butanol. Neutral glycosides are characterized by some differences; after the addition of glucose to C-3, a complication of the structure has been observed as a result of the introduction of additional glucose fragments at C-2′, galactose, glucose, di- and tri-glucosyl fragments at C-3′, and also glucose, galactose, and a di-galactosyl moiety at C-4′. At position C-28 of oleanolic acid, only glucose can exist.

Glycosides of other triterpene acids (e.g., morolic acid (**323**), moronic acid (**325**), echinocystic acid (**327**), cochalic acid (**332**), machaerinic acid (**335**), and mesembryanthemoidigenic acid (**338**)) are both neutral and/or acidic derivatives.

In *C. officinalis*, two compounds related to rare 3,4-*seco*-terpene alcohols, which are derivatives of tirucallan (3,4-*seco*-cucurbitane or 3,4-*seco*-19(10→9)*abeo*-euphane), have been identified as helianol (**341**) and thirucalla-7,24-dienol (**342**) [3]. Previously, both compounds were found in tubular flowers of *Helianthus annus* L. [110].

The most distributed triterpene glycoside is glucoside D (**295**), which has been found in four species: *C. arvensis*, *C. officinalis*, *C. stellata*, and *C. suffruticosa*. Three species (*C. arvensis*, *C. officinalis*, *C. stellata*) contain glucoside C (**300**), calenduloside C (**312**), and calenduloside D (**320**), and seven glycosides (**293**, **299**, **303**, **309**, **318**, **319**, **333**) were identified in two species. Triterpenoids are quantitatively the main group of *Calendula* metabolites, which reaches up to 3–4% of the total level of fatty esters of faradiol, arnidiol, and calenduladiol [58], and up to 9% of triterpenoid glycosides [111].

Scientometric studies have shown a number of mismatches in the names of some triterpenoid glycosides; specifically, for individual compounds, several trivial names are used. For the first time, six glycosides of oleanolic acid (containing a glucuronic acid residue at the C-3 position of the aglycone) were isolated from the flowers of *C. officinalis* and characterized by Kasprzyk Z. and Wojciechowski Z. in 1967, giving them the names glucosides A (**291**), B (**293**), C (**295**), D (**296**), E (**300**), and F (**303**) [67]. Later, Wojciechowski Z. et al. (1971) established the existence of a second group of oleanolic acid glycosides in *C. officinalis* containing a glucose residue at the C-3 position of the aglycone, named glucosides I (**308**), II (**310**), III (**311**), IV (**313**), V (**314**), VI (**315**), VII (**316**), and VIII (**321**) [71]. The latter research group used other names for glycosides A–F, such as glucuronides A–F, which are still relevant [112]. Therefore, the question of the priority of names for compounds **291**, **293**, **295**, **296**, **300**, and **303** remains open; the use of both variants is legitimate. Of note, the variants of names for glucosides C (**295**), D (**296**), and F (**303**), such as calendulosides H, G, and E, respectively, proposed by Vecherko L.P. et al., who isolated these compounds from *C. officinalis* in 1975–1976 [69,74,76,79,80,81], can be considered as synonyms. Calenduloside F (**299**) was isolated and characterized by Vecherko L.P. et al. (1975) [76]; however, the final identification of this compound under the name glucoside D_2_ was performed by Vidal-Oliver E. (1989) [70]. Later, compound **299** was also named glucuronide D_2_ [9].

### 3.5. Carotenoids

Since the discovery of carotene, lycopene, and violaxanthin in pigmented marigold petals [27], approximately a hundred carotenoids (**343**–**437**) have been found and identified in *C. officinalis*. Only this species was studied for this group of compounds. Carotenoids have been found in free and esterified forms, including myristic, palmitic, and stearic acid mono- and di-esters [85]. The most diverse carotenoid aglycone is lutein, which forms 32 compounds (**376**–**407**), followed by violaxanthin (**418**–**428**), cryptoxanthin (**362**–**369**), and zeaxanthin (**429**–**434**). Owing to the wide variety of colors of calendula flowers (ranging from white to burgundy and maroon), different varieties have different levels of carotenoids, ranging from trace amounts to 200 mg per 100 g of dry flower petals [113,114].

### 3.6. Phenols

**Figure 5:**
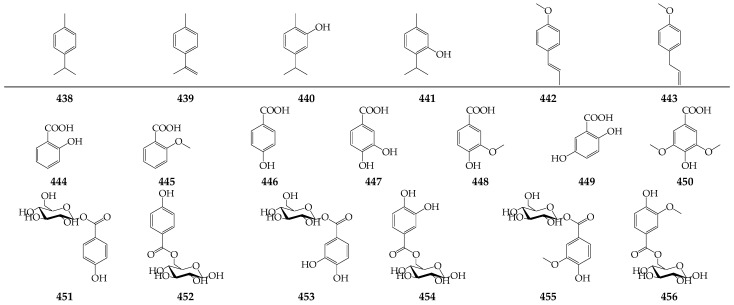
Phenols **438**–**443** and benzoic acid derivatives **444**–**456**.

Six simple phenols (i.e., *p*-cymene (**438**), *p*-cymenene (**439**), carvacrol (**440**), thymol (**441**), *p*-anethole (**442**), and estragole (**443**)) are the minor constituents of the essential oil of *C. officinalis* [30,35,36] and *C. arvensis* [16] (Figure 5).

### 3.7. Benzoic Acid Derivatives

Seven simple benzoic acids were identified as minor components of methanolic and ethanolic extracts of *C. officinalis* flowers, including salicylic acid (**444**), *o*-anisic acid (**445**), *p*-hydroxybenzoic acid (**446**), protocatechuic acid (**447**), vanillic acid (**448**), gentisic acid (**449**), and syringic acid (**450**) [35,87,89] (Figure 5). Later, six glucosides of *p*-hydroxybenzoic acid (**451**, **452**), protocatechuic acid (**453**, **454**), and vanillic acid (**455**, **456**) were identified in leaves and pollen of *C. officinalis* [90,91].

### 3.8. Hydroxycinnamates

**Figure 6:**
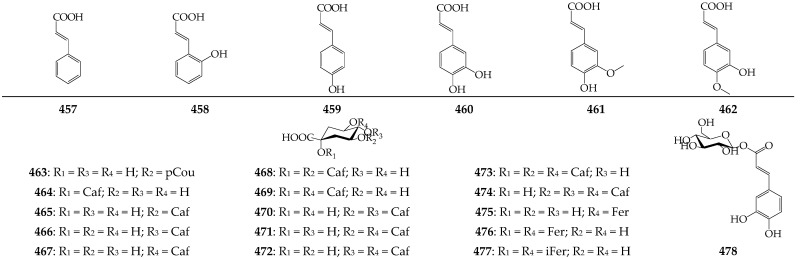
Hydroxycinnamates **457**–**478**. Caf—caffeoyl; pCou—*p*-coumaroyl; Fer—feruloyl; iFer—isoferuloyl.

Twenty two derivatives of cinnamic acid of *Calendula* genus (i.e., cinnamic acid (**457**), coumaric acids (**458**, **459**), caffeic acid (**460**), ferulic acid (**461**), isoferulic acid (**462**), mono-*O*-caffeoyl quinic acids (**464**–**467**), di-*O*-caffeoyl quinic acids (**468**–**472**), tri-*O*-caffeoyl quinic acids (**473**, **474**), 5-*O*-feruloylquinic acid (**475**), 1,5-di-*O*-feruloylquinic acid (**476**), 1,5-di-*O*-isoferuloylquinic acid (**477**), and 1-*O*-caffeoyl glucose (**478**)) were identified in the herb, roots, and pollen of *C. arvensis*, *C. officinalis*, *C. suffruticosa*, and *C. tripterocarpa* [75,89,92] (Figure 6).

Hydroxycinnamates are typical metabolites of asteraceous plants [115]; therefore, it is not surprising that they have been identified in calendulas. The dominant hydroxycinnamates in the flowers (3-*O*-caffeoylquinic acid (**465**) and 3,5-di-*O*-caffeoyl quinic acid (**471**)) amounted to 1–7 mg/g for **465** and 0.5–2 mg/g for **471**; while in the leaves, the content of **465** can reach 9 mg/g [89].

### 3.9. Coumarins

**Figure 7:**
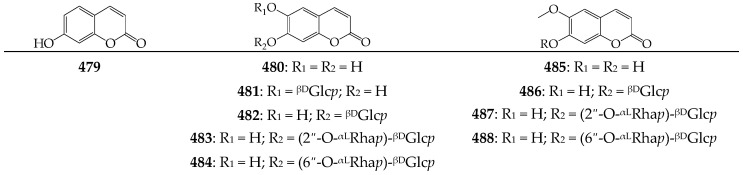
Coumarins **479**–**488**. ^βD^Glc*p*—β-D-glucopyranose; ^αL^Rha*p*—α-L-rhamnopyranose.

A small group of α-pyrone compounds or coumarins (ten compounds (**479**–**488**)) has been identified in small amounts in the flowers, leaves, and herb of *C. officinalis* [90,94,95] and *C. tripterocarpa* [92], including umbelliferone (**479**), esculetin (**480**) and glycosides (**481**–**484**), scopoletin (**485**), and glycosides (**486**–**488**) (Figure 7). The carbohydrate moieties of glycosides contain a glycose in esculin (**481**), cichoriin (**482**), and scopolin (**486**), neohesperidose in neoisobaisseoside (**483**) and haploperoside D (**487**), and rutinose in haploperoside (**484**) and isobaisseoside (**488**).

### 3.10. Flavonoids and Anthocyanins

**Figure 8:**
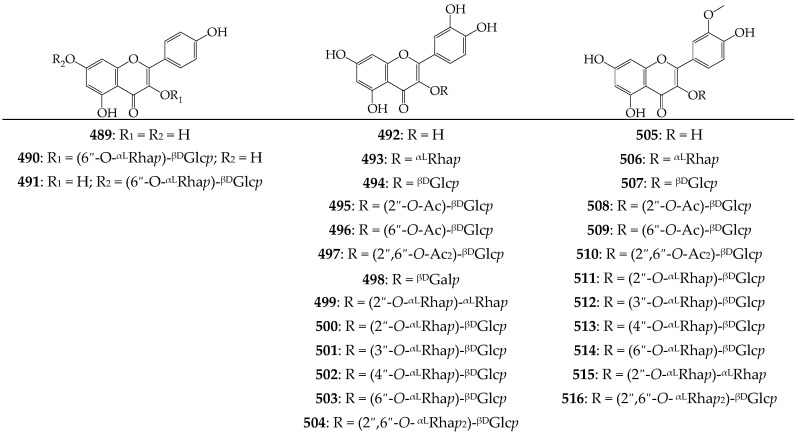
Flavonoids **489**–**516**. Ac—acetyl; ^βD^Gal*p*—β-D-galactopyranose; ^βD^Glc*p*—β-D-glucopyranose; ^αL^Rha*p*—α-L-rhamnopyranose.

Since the discovery of isorhamnetin (**505**), isorhamnetin-3-*O*-glucoside (**507**), and narcissin (**514**) in *C. officinalis* flowers in 1962 [116,117], twenty-eight flavonoids of *C. arvensis*, *C. officinalis*, *C. stellata*, *C. suffruticosa*, and *C. tripterocarpa* were also identified; the glycosyl derivatives of kaempferol (**489**–**491**), quercetin (**492**–**504**), and isorhamnetin (**505**–**516**) are the predominant forms of flavonoids (Figure 8). Carbohydrate fragments may exist as *monosaccharides* (incorporate one moiety of rhamnose, galactose, and glucose), *disaccharides* (including neohesperidose (2-*O*-ramnosylglucose), such as calendoflavobioside (**500**) and calendoflavoside (**511**) [97]; rungiose (3-*O*-ramnosylglucose) such as calendoside II (**501**) and IV (**512**) [91]; 4-*O*-ramnosylglucose, such as calendoside I (**502**) and III (**513**) [91]; rutinose (6-*O*-ramnosylglucose), such as nicotiflorin (**490**), kaempferol-7-*O*-rutinoside (**491**), rutin (**503**), and narcissin (**514**) [93,97,98]; and 2-*O*-ramnosylrhamnose, such as quercetin-3-O-(2″-*O*-ramnosyl)-rhamnoside (**499**) and calendoflaside (**515**) [97]), and *trisaccharides* (2,6-di-*O*-ramnosylglucose in manghaslin (**504**) and thyphaneoside (**516**) [98,100]). Monoglucosides of quercetin and isorhamnetin may sometimes be acylated by acetic acid giving mono- (**495**, **496**, **508**, **509**) or diacetates (**497**, **510**) [89,91]. The content of flavonoids in different parts varies from trace amounts in the roots and seeds to 2–4% in the tubular and ligular flowers; isorhamnetin derivatives are typically the major components [89,114]. Anthocyanins **517**–**524**, as components of red colored marigold ray florets, are glycosides of cyanidin, delphinidin, malvidin, paeonidin, pelargonidin, and petunidin with a total content of 0.6–1.2% [89].

### 3.11. Other Compounds

Highly lipophilic compounds found in essential oils and hexane fractions of *C. arvensis*, *C. officinalis*, and *C. suffruticosa* include alkanes (**525**–**550**), aliphatic alcohols (**551**–**559**), aliphatic aldehydes and ketones (**560**–**565**), fatty acids and esters (**566**–**602**), and chromanols (**603**–**613**) [33,34,36,39]. In methanolic and water extracts of *Calendula* species, various hydrophilic compounds have been identified, including organic acids (**614**–**616**), carbohydrates (**617**–**630**), and amino acids (**631**–**646**) [41,108]. In essential oils of *C. officinalis*, 3-cyclohexene-1-ol, 3-cyclohexene-1-ol 4-methyl ester, loliolide, 1,2,3,5,8,8α-hexahydronaphthalene 6,7-dimethyl ester, 4-methylacethophenone, and 1-methyl ethyl hexadecanoate were identified [30,31,35,109]; tricyclene, 1H-benzocyclohepten-9-ol, and 2-pentyl furane were identified in *C. arvensis* [16,33,41]; naphthalene was detected in *C. suffruticosa* [42].

### 3.12. Polysaccharides

The study of *Calendula* polysaccharides started in the mid-1980s [118] and refers only to *C. officinalis* flowers; none of the other species have been studied (Table 3). A group of German researchers conducted a systematic study of plant polysaccharides and their immunostimulating properties [8]. After the 0.5 M NaOH extraction of *C. officinalis* flowers, three neutral polysaccharides were isolated and characterized as rhamnoarabino-3,6-galactan and two arabino-3,6-galactans [119].

Later, five water-soluble polymers with 24.1–57.2 mol% of uronic acids were identified and demonstrated a wide variation of arabinose (4.0–12.5 mol%) and galactose (14.1–40.8 mol%) levels [120]. Polysaccharide fractions were also isolated from the industrial *C. officinalis* flower wastes; a high uronic content was typical for them (58.3–64.0 mol%) as well as variation in the level of neutral monosaccharides [121,122]. The exact structure of the acidic polysaccharides of *C. officinalis* is still unknown.

## 4. Separation of *Calendula* Metabolites by GC and LC

The chemical characteristics and chromatographic properties of the *Calendula* metabolites determine which technique is used to achieve satisfactory separation of target compounds. Some differences exist between gas chromatography and liquid chromatography (LC) methods designed for analyzing sterols, triterpenes, carotenoids, fatty acids, and phenolic compounds that are found in *Calendula* plants (Table 4).

### 4.1. Sterols

Both GC and LC techniques were designed to separate sterols with various structures. Various 30 m columns (e.g., ZB-1 [123], HP-5MS UI [124], DB 17 [3], and RTX^®^-1 MS [56]) were used to analyze sterol alcohols and esters by GC with flame ionization detection (GC-FID) and mass spectrometric detection (GC-MS). Fatty acid esters of arnitriol, faradiol, arnidiol, and maniladiol demonstrated appropriate LC separation on 250 mm reversed-phase (RP) columns (e.g., LiChrosphere RP-8 [125] and RP-18e [126], Hypersil ODS [60], Nucleosil 100-5 C18 [58], and Superiorex ODS C18 [3]) using isocratic elution with methanol [3,60,125], a water–methanol mixture [58], as well as gradient elution with trifluoroacetic acid–methanol mixtures [126,127,128] and ultraviolet (UV) or diode array (DAD) detection at 210 nm. Shorter columns (e.g., Kinetex C18 (100 mm) and Kromasil 100Å (50 mm)) showed good separation of 10 sterol esters by LC with atmospheric pressure chemical ionization quadrupole time-of-flight mass spectrometric detection (LC-APCI-QTOF-MS) [56].

### 4.2. Triterpenes and Glycosides

Aglycones (oleanolic acids) and glycosides were analyzed using high-performance liquid chromatography with UV (HPLC-UV) and mass-spectrometric detection (HPLC-UV-MS) assays using 250 mm (KromaPhase C18 [129], Eurospher 100 C18 [130]) and 150 mm RP columns (Waters Sunfire RP C_18_ [111], C18 Luna [131]) with isocratic [129] or gradient elution in mixtures of acetic acid and acetonitrile [111,130,131]. Detection at 205–215 nm and MS detection in negative ionization mode allowed the analysis of two to six components [111,129,130,131].

### 4.3. Carotenoids

The chromatographic separation of *Calendula* carotenoids was realized using HPLC with diode-array detection (DAD) and HPLC-UV-MS techniques. To qualitatively and quantitatively analyze carotenes, lutein, lycopene, and other pigments, the RP sorbents are traditionally used in 250 mm and in 300 mm columns (e.g., C30 YMC [85], Nucleosil ODS C18 [86], YMC [132], Bondclone C18 [133], Nucleodur C18 [134], and Inertsil ODS-3 C18 [135]). Isocratic elution (with methanol–acetonitrile–methylene chloride–cyclohexene [133], acetone–water [134], methanol–tetrahydrofuran–water [135], and acetonitrile–methanol [136] mixtures) and gradient elution (with acetonitrile–water–ethyl acetate [86] and methanol–methyl *tert*-butyl ester–water [85,132] mixtures) were successfully performed. The strong absorption of carotenoids in the visible spectral region allowed their detection at 450–474 nm wavelengths [132,133,134,135,136] as well as by MS detection using atmospheric pressure chemical ionization (APCI) [85].

### 4.4. Fatty Acids

The fatty acid composition of *C. officinalis* seeds was extensively studied by GC assays on BPx-70 (60 m) [105], DB-23 (30 m) [137], HP-88 (100 m) [138], and Supelco SP-2560 (100 m) [139] columns and resulted in the quantification of 7–17 compounds with electron impact [105,137,138] and chemical ionization [139] MS detection.

**Table 4:** Synopsis of the methods of *Calendula* extracts analysis, separation conditions, detectors, and separated compounds.

Assay ^a^, Ref.	Separation Conditions ^b^	Detection	Compounds
Sterols			
GC-FID [123]	C: Zebron ZB-1 (30 m × 0.25 mm, 0.25 µm; Phenomenex, Torrans, CA, USA)	MS: FID	Oleanolic acid, campesterol, cholesterol, isofucosterol, 24-methylenecycloartanol, sitosterol, sitostanol, stigmasterol, stigmast-7-en-3-ol
GC-MS/FID [124]	C: HP-5MS UI (30 m × 0.25 mm, 0.25-μm; Agilent Technologies, Santa-Clara, CA, USA)	MS: FID	Oleanolic acid, campesterol, cholesterol, isofucosterol, sitosterol, sitostanol, stigmasterol, tremulone, 24-methylenecycloartanol
GC-MS [3]	C: DB 17 (30 m × 0.3 mm; Agilent Technologies, Santa-Clara, CA, USA)	MS: ESI (70 eV)	Helianol; taraxerol; dammaradienol; α/β-amyrins; cycloartenol; tirucalla-7,24-dienol; lupeol; 24-methylene-cycloartanol; ψ-taraxasterol, taraxasterol
GC-MS [56]	C: RTX^®^-1 MS (30 m × 0.25 mm; Restek, Cartersville, GE, USA)	MS: EI (70 eV)	3-*O*-Palmitates and 3-*O*-myristates of arnidiol, arnitriol A, faradiol, lupane-3β,16β,20-triol, and maniladiol
HPLC-UV [125]	C: LiChrosphere RP-8 (250 × 15 mm, 5 μm; Merck, Kenilworth, NJ, USA); I; E: MeOH	UV: λ 210 nm	3-*O*-Palmitate and 3-*O*-myristate of faradiol
HPLC-UV [126,127,128]	C: LiChrosphere RP-18e (250 × 4 mm, 5 μm; Merck, Kenilworth, NJ, USA); G; E: TFA (A), MeOH (B); 0–50 min 95–100 %B, 50–95 min 100 %B; T 25 °C; ν 1.5 mL/min	UV: λ 210 nm	3-*O*-Palmitate, 3-*O*-myristate and 3-*O*-laurate of faradiol
HPLC-DAD [60]	C: Hypersil ODS (250 × 4.6 mm, 5 μm; Thermo Fisher Scientific, Waltham, MA, USA); I; E: MeOH; ν 1 mL/min	DAD: λ 210 nm	3-*O*-Palmitates, 3-*O*-myristates and 3-*O*-laurates of faradiol and maniladiol; taraxasterol, β-amyrin
HPLC-UV [58]	C: Nucleosil 100-5 C18 (250 × 4 mm, 5 μm; Macherey-Nagel, Düren, Germany); I; E: MeOH-H_2_O 97:3; ν 1.5 mL/min	UV: λ 210 nm	3-*O*-Palmitates, 3-*O*-myristates and 3-*O*-laurates of arnidiol, faradiol and calenduladiol
HPLC-UV [3]	C: Superiorex ODS C18 (250 × 10 mm, 5 μm; Osaka Soda, Osaka, Japan); I; E: MeOH; ν 4 mL/min	UV: λ 210 nm	Helianol; taraxerol; dammaradienol; α/β-amyrins; cycloartenol; tirucalla-7,24-dienol; lupeol; 24-methylene-cycloartanol; ψ-taraxasterol, taraxasterol
LC-APCI-QTOF-MS [56]	1. C: Kinetex C18 (100 × 3 mm, 2.6 µm; Phenomenex, Torrans, CA, USA); G; E: MeCN (A), MeOH (B); 0–1 min 0%B, 1–10 min 0–100%B, 10–15 min 100%B; ν 400 µL/min 2. C: Kromasil 100Å (50 × 4 mm, 5 µm; Kromasil, Göteborg, Sweden); G; E: MeOH (A), *i*-PrOH (B); 0–1 min 30%B, 1–25 min 30–100%B, 25–30 min 100%B; ν 1.2 mL/min	MS: CE	3-*O*-Palmitates and 3-*O*-myristates of arnidiol, arnitriol A, faradiol, lupane-3β,16β,20-triol, and maniladiol
Triterpenes and Glycosides			
HPLC-UV [129]	C: KromaPhase C18 (250 mm × 4.6, 5 µm; Kromasil, Göteborg, Sweden); I; E: MeCN-H_2_O 90:10; ν 1 mL/min	UV: λ 210 nm	Oleanolic acid
HPLC-UV-MS [130]	C: Eurospher 100 C18 (250 × 4 mm, 5 µm; Knauer, Berlin, Germany); G; E: 0.5% CH_3_COOH in MeCN (A), 0.5% CH_3_COOH in H_2_O (B); 1–15 min 20% A, 15–45 min 46% A, 45–90 min 55% A, 90–100 min 90% A, 100–110 min 20% A; ν 0.6 mL/min	UV: λ 210 nm; MS: neg.	Glycosides A, B; calendulosides H, F, G, E
HPLC-UV-MS [111]	C: Waters Sunfire RP C_18_ (150 × 2.1 mm, 5 µm; Waters, Milford, MA, USA); G; E: 0.12% CH_3_COOH in 10% MeCN (A), 0.12% CH_3_COOH in 100% MeCN (B); 0–3 min 75% A, 3–25 min 75–50% A, 25–28 min 50–25% A, 28–33 min 100% B; ν 0.2 mL/min	UV: λ 205, 215 nm; MS: neg.	Glycosides A, B, C, D, D_2_
HPLC-UV-MS [131]	C: C18 Luna (150 × 4.6, 5 μm; Phenomenex, Torrans, CA, USA); G; E: H_2_O (A), MeCN (B), CH_3_COOH in 10% MeCN (C); 0–47 min 90%A-O%B-10%C→43%A-47%B-10%C, 0–47 min 0%A-90%B-10%C	UV: λ 210 nm; MS: neg.	Glycosides A, B, C, D, F; calenduloside A
Carotenoids			
HPLC-DAD [86]	C: Nucleosil ODS C18 (250 × 4.6 mm, 5 µm; Macherey-Nagel, Düren, Germany); G; E: MeCN-H_2_O 9:1 in 0.25% TEA (A), EtOAc in 0.25% TEA (B); 0–10 min 90–50% A, 10–20 min 50–10% A; ν 1 mL/min	DAD: λ 450 nm	Antheraxanthin, carotene (α-, β-, γ-), flavoxanthin, lactucaxanthin, lutein, lycopene, mutatoxanthin, (9*Z*)-neoxanthin, rubixanthin, zeaxanthin
HPLC-DAD [132]	C: YMC (250 × 4.6 mm, 5 µm; YMC Co., Kyoto, Japan); G; E: MeOH-MTBE-H_2_O 90:6:4 (A); MeOH-MTBE-H_2_O 25:71:4 (B); 0–12 min 100% A, 12–96 min 0% A; ν 1 mL/min	DAD: λ 450 nm	γ-Carotene, lycopene, rubixanthin
HPLC-DAD [133]	C: Bondclone C18 (300 × 3.9 mm, 10 µm; Phenomenex, Torrans, CA, USA); I; E: MeOH-MeCN-MeCl-cyclohexene 22:55:11.5:11.5; ν 0.8 mL/min	DAD: λ 440 nm	β-Carotene, lutein
HPLC-DAD [134]	C: Nucleodur C18 (250 × 4.6 mm, 5 µm; Macherey-Nagel, Düren, Germany); I; E: H_2_O-Me_2_CO 13:87; ν 1 mL/min	DAD: λ 445 nm	Lutein, zeaxanthin
HPLC-DAD [135]	C: Inertsil ODS-3 C18 (250 × 4.6 mm; GL Sciences, Torrance, CA; USA); I; E: MeOH-THF-H_2_O 37:60:3; ν 1.4 mL/min	DAD: λ 474 nm	Astaxanthin, canthaxanthin, β-carotene
HPLC-DAD [136]	C: C18 (250 × 4.6 mm, 5 µm); I; E: MeCN-MeOH 40:60; ν 1 mL/min	DAD: λ 446 nm	Lutein
HPLC-DAD-MS [85]	C: C30 YMC column (250 × 4.6 mm, 5 μm; YMC Co., Kyoto, Japan); G; E: MeOH-MTBE-H_2_O 81:15:4 (A), MeOH-MTBE-H_2_O 16:80.4:3.6 (B); 0–39 min 99–44% A, 39–45 min 44–0% A; ν 1.0 mL/min	DAD: 450 nm MS: APCI	74 Compounds
Fatty Acids			
GC-MS [105]	C: BPx-70 (60 m × 0.25 mm, 0.25 µm; Trajan Scientific and Medical, Victoria, Australia)	MS: EI (70 eV)	11 Acids
GC-MS [137]	C: DB-23 (30 m × 0.25 mm, 0.25 μm; Agilent Technologies, Santa-Clara, CA, USA)	MS: EI (70 eV)	12 Acids
GC-MS [138]	C: HP-88 (100 m × 25 mm, 0.2 µm; Agilent Technologies, Santa-Clara, CA, USA)	MS: EI (70 eV)	7 Acids
GC-MS [139]	C: Supelco SP-2560 (100 m × 0.25 mm, 0.2 µm; Sigma-Aldrich, Saint Louis, MI, USA)	MS: CI	17 Acids
Phenolic Compounds			
HPLC-UV [140]	C: SiliaChrom C-18 (150 × 4.6 mm, 5 µm; SiliCycle, Quebec, Canada); G; E: 0.08% H_3_PO_4_ (A), MeOH (B); 0–1.5 min 35% B, 1.5–4 min 35–50% B, 4–12 min 55% B, 12–13 min 50–100% B, 13–20 min 100% B, 20–21 min 100–35% B, 21–30 min 35% B; ν 1 mL/min	UV: λ 370 nm	Quercetin
HPLC-UV [141]	C: Hypersyl C18 (250 × 4.6 mm, 5 µm; Thermo Fisher Scientific, Waltham, MA, USA); I; E: MeCN-2% CH_3_COOH in H_2_O 15:85; ν 1 mL/min	UV: λ 340 nm	Narcissin, rutin
HPLC-UV [142]	C: Phenomenex C18 (100 × 4.6 mm, 5 µm; Phenomenex, Torrance, CA, USA); I; E: MeCN-2% HCOOH 15:85; ν: 0.5 mL/min	UV: λ 254 nm	Chlorogenic, caffeic acids, rutin
HPLC-UV [143]	C: Zorbax SB-C18 (100 × 3 mm, 3.5 µm; Agilent Technologies, Santa-Clara, CA, USA); G; E: 0.1% HCOOH in H_2_O (A), MeOH (B); 0–35 min 5–42% B; ν 1 mL/min; T 48 °C	UV: λ 330, 370 nm	Caffeic, chlorogenic, *p*-coumaric, ferulic acids, isoquercitrin, rutin, quercetin
HPLC-UV [96]	K: Schim-pack C-18 (250 × 4.6 mm, 5 µm; Shimadzu, Columbia, MA, USA); G; E: 0.1% HCOOH in H_2_O (A), 0.1% HCOOH in MeCN (B); 0–1 min 5% B, 1–12 min 5–100% B, 12–16 min 100% B, 16–18 min 100–5% B; ν 200 µL/min	UV: λ 280, 335 nm	Isoquercitrin, isorhamnetin, isorhamnetin-3-*O*-glucoside, rutin, scopolin
HPLC-UV [133]	C: Bondclone C18 (300 × 3.9 mm, 10 µm; Phenomenex, Torrance, CA, USA); G; E: 15% CH_3_COOH in H_2_O (A), MeOH (B); 0–15 min 5% B; ν 1.5 mL/min	UV: λ 254 nm	Isoquercitrin, narcissin, quercetin, scopolin
HPLC-UV [90,114,144]	C: ProntoSIL-120-5-C18 AQ (75 × 2 mm, 5 µm; Knauer, Berlin, Germany); G; E: 0.2 M LiClO_4_ in 0.006 M HClO_4_ (A), MeCN (B); 0–7.5 min 11–18% B, 7.5–13.5 min 18% B, 13.5–15 min 18–20% B, 15–18 min 20–25% B, 18–24 min 25% B, 24–30 min 25–100% B; ν: 150 µL/min; T 35 °C	UV: λ 270 nm	3-*O*-Caffeoylquinic, caffeic acids, thyphaneoside, isoquercitrin, rutin, quercetin-3-*O*-(6″-acetyl)-β-d-glycoside, 3,5-di-*O*-caffeoylquinic, 1,5-di-*O*-caffeoylquinic, 4,5-di-*O*-caffeoylquinic acids, isorhamnetin-3-*O*-β-d-glucoside, isorhamnetin-3-*O*-(6″-acetyl)-β-d-glycoside
HPLC-PDA [145]	C: X-Bridge C18 (250 × 4.6 mm, 5 µm; Waters, Milford, MA, USA); I; E: MeCN-MeOH-H_2_O 30:2:68; ν: 0.5 mL/min	PDA: λ 254 nm	Rutin
HPLC-DAD [146]	C: Eclipse XDB-C18 (150 × 4.6 mm, 5 µm; Agilent Technologies, Santa-Clara, CA, USA); G; E: 0.1% H_3_PO_4_ in MeOH (A), 0.1% H_3_PO_4_ in iPrOH (B); 0–10 min 10–15% B, 10–20 min 15–20% B	DAD: λ 280, 330 nm	Caffeic, chlorogenic, vanilic, *p*-coumaric, *t*-2-hydroxycinnamic acids
HPLC-DAD [147]	C: ODS Hypersil C18 (250 × 4.6 mm, 5 µm; Thermo Fisher Scientific, Waltham, MA, USA); G; E: 0.33 M CH_3_COOH (A), MeOH (B); 0–80 min 8–70% B; ν 80 µL/min	DAD: λ 327, 356 nm	Quercetin, rutin
HPLC-DAD [148]	C: Phenomenex C18 (250 × 4.6 mm, 5 μm; Phenomenex, Torrance, CA); G; E: 0.5% CH_3_COOH (A), MeOH (B); 0–2 min 1–5% B, 2–10 min 5–20% B, 10–40 min 20–45% B, 40–55 min 70% B, 55–75 min 100% B; ν 0.6 mL/min	DAD: λ 327, 366 nm	Chlorogenic, caffeic, rutin, quercetin, kaempferol
HPLC-DAD [149]	C: Spherisorb S3 ODS-2 C18 (150 × 4.6 mm, 3 µm); G; E: 0.1% HCOOH (A), MeCN (B); 0–5 min 15% B, 5–10 min 15–20% B, 10–20 min 20–25% B, 20–30 min 25–35% B, 30–40 min 35–50% B	DAD: λ 280, 370 nm	5-*O*-Caffeoylquinic acid, quercetin-3-*O*-rhamnosylrutinoside, quercetin-3-*O*-rutinoside, kaempferol-*O*-rhamnosylrutinoside, isorhamnetin-3-*O*-rhamnosylrutinoside, isorhamnetin-3-*O*-neohesperidoside, quercetin-3-*O*-(6″-acetyl)-glucoside, isorhamnetin-3-*O*-rutinoside, isorhamnetin-3-*O*-glucoside, isorhamnetin-3-*O*-(6″-acetyl)-glucoside
HPLC-DAD [150]	C: Phenomenex Kinetex Phenyl-hexyl (150 × 4.6 mm, 2.6 μm; Phenomenex, Torrance, CA); G; E: 0.1% HCOOH (A), 0.1% HCOOH in MeCN (B); 0–5 min 10% B, 5–35 min 15–45 % B, 35–40 min 45–100 % B; ν 500 μL/min	DAD: λ 330 nm	Chlorogenic acid, thyphaneoside, manghaslin, rutin, calendoflavoside, narcissin
HPLC- UV-MS [151]	C: RP Zorbax Eclipse Plus C18 (150 × 4.6 mm, 1.8 µm; Agilent Technologies, Santa-Clara, CA, USA); G; E: 0.2% HCOOH in H_2_O (A), MeCN (B); 0–3 min 5–24% B, 3–6 min 24% B, 6–24 min 24–38% B, 24–30 min 38–99% B, 30–33 min 99% B, 33–34 min 99–5% B; ν 0.8 mL/min	UV: λ 356 nm MS: neg.	3-*O*-Caffeoylquinic acid, isorhamnetin-3-*O*-glucoside, isorhamnetin-3-*O*-acetylglucoside, manghaslin, narcissin, rutin, thyphaneoside
HPLC- UV-MS [152]	C: Aquapore RP-300 (220 × 4.6 mm, 5 µm; PerkinElmer, Waltham, MA, USA); I; E: iPrOH-THF-CH_3_COONH_4_ pH 4.5 10:5:85; ν 1.2 mL/min	UV: λ 360 nm MS: neg.	Thyphaneoside
HPLC- UV-MS [100,153]	C: LiChrosorb RP18 (10 × 4 mm, 5 µm; Merck, Kenilworth, NJ, USA); G; E: MeCN (A), phosphate buffer pH 3.0 (B); 0–10 min 12% B, 10–15 min 12–18% B, 15–30 min 18–45% B, 30–42 min 45–100% B, 42–50 min 100–12% B; ν 1.3 mL/min; T 26 °C	UV: λ 254, 330, 350 nm MS: neg.	3-*O*-Caffeoylquinic acid, isoquercitrin, isorhamnetin-3-*O*-glucoside, isorhamnetin-3-*O*-acetylglucoside, manghaslin, narcissin, rutin, thyphaneoside
HPLC- UV-MS [131]	C: C18 Luna (150 × 4.6 mm, 5 μm; Phenomenex, Torrans, CA, USA); G; E: H_2_O (A), MeCN (B), CH_3_COOH in 10% MeCN (C); 0–47 min 90%A-0%B-10%C→43%A-47%B-10%C, 0–47 min 0%A-90%B-10%C	UV: λ 254 nm; MS: neg.	Narcissin, thyphaneoside
HPLC- UV-MS [75]	C: Hypersil gold column (1000 × 20 mm, 1.9 µm; Thermo Fisher Scientific, Waltham, MA, USA); G; MeCN (A), 0.1% HCOOH (B); 0–14 min 5% B, 14–16 min 5–40 % B, 16–23 min 40–100 % B, 23–33 min 100–5 % B; ν 0.2 mL/min; T 30 °C	UV: λ 280 nm; MS: neg.	40 Compounds
UHPLC-DAD [154]	C: Acquity UPLC HSS T3 (150 × 2.1 mm, 1.8 µm; Waters, Milford, MA, USA); G; E: H_2_O (A), MeCN (B); 0.0–4.0 min 3–13% B, 4.0–5.0 min 13–17.5% B, 5.0–9.0 min 17.5% B, 9.0–12.5 min 17.5–24.5% B, 12.5–17.0 min 24.5–30.0% B, 17.0–25.0 min 30.0% B, 25.0 min 3.0% B, 25.0–30.0 min 3.0% B; ν 275 µL/min	UV: λ 330 nm	Chlorogenic acid, typhaneoside, narcissin

^a^ Assay: APCI-QTOF—atmospheric pressure chemical ionization quadrupole time-of-flight; DAD—diode array detector; FID—flame ionization detector; GC—gas chromatography; HPLC—high-performance liquid chromatography; MS—mass spectrometric detector; PDA—photodiode arrary detector; UHPLC—ultra high-pressure liquid chromatography; UV—ultraviolet. ^b^ Separation conditions: column (C); elution mode (I—isocratic, G—gradient); eluents (E; iPrOH—isopropanol; MeCN—acetonitrile; MTBE—methyl *tert*-butyl ester; THF—tetrahydrofuran); column temperature (T).

### 4.5. Phenolic Compounds

Evaluation of phenolic compounds in *Calendula* plants is an important task, as indicated by the known HPLC protocols found in the scientific literature. To separate target compounds, only RP C18 columns with varying lengths were used, such as 75 mm ProntoSIL-120-5-C18 [90,114,144]; 100 mm Phenomenex C18 [142], Zorbax SB-C18 [143], and LiChrosorb RP18 [100,153]; 150 mm Luna C18 [131], SiliaChrom C-18 [140], Eclipse XDB-C18 [146], Spherisorb S3 ODS-2 C18 [149], Zorbax Eclipse Plus C18 [151], and Aquity UPLC HSS T3 [154]; 220 mm Aquapore RP-300 [152]; 250 mm Shim-pack C-18 [96], Hypersil C18 [141,147], X-Bridge C18 [145], and Phenomenex C18 [148]; 300 mm Bondclone C18 [133]; and 1000 mm Hypersil Gold [75]. The presence of various eluents requires the frequent use of formic acid [75,96,142,150,151], acetic acid [133,141,148], phosphoric acid [140,146] as the polar eluent and methanol [133,140] and acetonitrile [96,114,141,142] as the non-polar eluent. The addition of lithium perchlorate [90,114,144] and tetrahydrofuran [152] resulted in better resolution and improved peak shapes. Detection in the region at 254–280 nm and/or 330–370 nm corresponds to the maximum absorption of most phenolic compounds. The optimized LC conditions resulted in the separation of basic flavonoids and hydroxycinnamates of *Calendula*.

## 5. Concluding Remarks and Future Perspectives of *Calendula* Metabolites Research

Based on the results of previous studies, for the genus *Calendula*, a situation has been observed that is typical for industrial plant species that are widely used in human life. For such species, knowledge is skewed in favor of a single plant that is a commercial product, such as *C. officinalis*, which is the only species from the genus that is widely used. An incomparably smaller amount of information is available for *C. arvensis*, *C. stellata*, *C. suffruticosa*, and *C. tripterocarpum*, and seven other species (*C. eckerleinii*, *C. karakalensis*, *C. lanzae*, *C. maroccana*, *C. meuselii*, *C. pachysperma*, *C. palaestina*) are still unstudied. Of note, *C. officinalis* is an example of the use of only one part of the plant (flowers) to the detriment of the rest of the biomass (leaves, stems, roots), which has been understudied and is typically wasted. Table 5 presents a synopsis of known knowledge and clearly demonstrates the current situation regarding the *Calendula* genus.

The actual situation in the field of studying *Calendula* chemodiversity indicates that essential oils of this genus are most often subjected to research. This occurs owing to the greater availability of instruments for this type of analysis, which is usually performed using the GC-MS technique, as well as the simplicity of sample preparation, which requires hydrodistillation (as the most common method of isolation). The same applies to the analysis of lipophilic extracts (hexane, dichloroethane, chloroform), which contain sterols, alkanes, aliphatic alcohols, aldehydes, ketones, and fatty acids. That is why there is an abundance of information on non-polar compounds. Of note, the lipophilic components of *Calendula* are currently of no practical importance; thus, excessive attention to them is not justified, at least until further studies are performed.

Sesquiterpene glycosides, unlike the sesquiterpene components of essential oils, have proven antiviral activity against a vesicular stomatitis virus (VSV) and rhinovirus (HRV type 1B) [47], antiprotozoal activity against *Leishmania donovani* [49], and anti-inflammatory activity [155]. However, the study of these valuable compounds is limited to only three species; in *C. officinalis*, only flowers have been studied; although, given the discovery of these compounds in the herb of *C. arvensis*, it would be worth paying attention to other parts of *C. officinalis*.

Researchers have made considerable progress in the study of triterpene alcohols, esters, and glycosides of *Calendula*. However, these studies refer primarily to *C. officinalis* from which 91 compounds have been isolated out of 109 known compounds. Compared to other compounds, for triterpenoid esters and glycosides, more in-depth pharmacological studies have been performed. Pharmacological studies demonstrated the anti-ulcer effect of calenduloside B (**319**) [156], antimutagenic activity of glycosides **291**, **295**, **296**, **299**, **300**, **303**, **309**, **312**, **318**, **320** [157], the anti-inflammatory activity of faradiol (**197**), lupeol (**189**) [6], and other triterpene alcohols [3] and some esters [125], hypoglycemic and gastroprotective potential of glucoside A (**303**), B (**296**), C (**300**), D (**295**), and F (**291**) [9], as well as their antibacterial, antiparasitic [158], and other activities. Owing to the clear potential of using triterpenoids as biologically active agents, it is necessary to expand the search for new compounds and new sources within the *Calendula* genus.

Phenolic compounds of the *Calendula* genus have been extensively studied; however, most of the scientific information related to *C. officinalis* does not allow global conclusions about the features of the phenolic distribution within the genus. The question of domination of only two flavonol aglycones (quercetin and isorhamnetin) in *Calendula* plants remains interesting and unexplored.

The studies of carotenoids, anthocyanins, and polysaccharides are limited to a single object, *C. officinalis* flowers, and these studies require more attention because of the availability and wide spectrum of bioactivity of these phytochemicals. Moreover, a detailed study of the fine stricture of polysaccharides of *C. officinalis* flowers is needed owing to the lack of information.

Because *C. officinalis* is an industrial plant, it is necessary to expand research on non-floral parts of the plant, such as leaves, stems, roots, and seeds. The volume of production of these parts of the plant must be gigantic, but there are currently no examples of their rational practical application. In terms of marigold pharmaceutic production, the waste from the industrial processing of *C. officinalis* flowers is not used as a resource for obtaining valuable products. Moreover, there are few examples of recycling waste from the pharmaceutical processing of plants. Currently, this wasteful approach can be regarded as irrational and requires more attention and reasonable proposals for processing plant waste.

In general, after almost a century of studying the genus *Calendula*, despite its widespread use, it is still the subject of numerous studies. Scientists are trying to expand the horizons of knowledge about its metabolites, application, and analysis because there are still many areas that need to be clarified. Taking into account the identified trends in the study of *Calendula*, we will still require scientific progress in the field of genus chemistry for a long period of time.

## Figures and Tables

**Table 1:** Review articles aimed at *Calendula* research.

Year	First Author, Title, Journal, Ref.	Total Count of Metabolites Referred
2006	Basch, E. et al. Marigold (*Calendula officinalis* L.): An evidence-based systematic review by the natural standard research collaboration. *J. Herb. Pharmacother*. [12]	17
2008	Leach, M.J. *Calendula officinalis* and wound healing: A systematic review. *Wounds* [13]	10
2009	Muley, B. et al. Phytochemical constituents and pharmacological activities of *Calendula officinalis* Linn (Asteraceae): A review. *Trop. J. Pharm. Res.* [14]	155
2010	Mishra, A. et al. *Calendula officinalis*: An important herb with valuable therapeutic dimensions—An overview. *J. Global Pharma Technol*. [15]	12
2013	Arora, D. et al. A review on phytochemistry and ethnopharmacological aspects of genus *Calendula*. *Pharmacogn. Rev*. [16]	92
2015	Kodiyan, A. et al. A review of the use of topical *Calendula* in the prevention and treatment of radiotherapy-induced skin reactions. *Antioxidants* [17]	-
2016	Ghédira, K. *Calendula officinalis* L. (Asteraceae): Souci. *Phytothérapie* [18]	67
2018	Cruceriu, D. et al. *Calendula officinalis*: Potential roles in cancer treatment and palliative care. *Integr. Cancer Ther.* [19]	4
2019	Chitrakar, B. et al. Edible flowers with the common name “marigold”: Their therapeutic values and processing. *Trends Food Sci. Technol.* [20]	17
2019	Givol, O. et al. A systematic review of *Calendula officinalis* extract for wound healing. *Wound Repair Regener.* [21]	-
2022	Abdelwahab, S.I. et al. Fifty-year of global research in *Calendula officinalis* L. (1971–2021): A bibliometric study. *Clin. Complement. Med. Pharmacol.* [22]	28
2022	Egeli, D. *Calendula officinalis* L. [23]	123

**Table 2:** Compounds **1**–**656** found in *Calendula* plants.

No	Compound ^a^	Species (organ) ^b^	Ref.
Monoterpenes			
**1**	Artemisia ketone	*C. officinalis* (f)	[29]
**2**	Bornyl acetate	*C. officinalis* (ae,l)	[30,31]
**3**	Camphene	*C. officinalis* (f)	[29]
**4**	Camphor	*C. officinalis* (f)	[29]
**5**	*δ*-3-Carene	*C. officinalis* (ae,l)	[30,31]
**6**	Carvenone	*C. officinalis* (f)	[29]
**7**	1,8-Cyneol	*C. officinalis* (ae,f,l)	[30,31]
**8**	β-Cyclocitral	*C. officinalis* (f)	[32]
**9**	*p*-Cymene	*C. arvensis* (ae)	[33]
**10**	Dihydrotagenone	*C. officinalis* (f)	[29]
**11**	Dill ether	*C. arvensis* (ae)	[34]
**12**	Geraniol	*C. officinalis* (ae)	[30]
**13**	Geranyl acetate	*C. arvensis* (ae)	[34]
**14**	Geranyl acetone	*C. officinalis* (f)	[32]
**15**	Linalool	*C. arvensis* (ae) *C. stellata* (f)	[16,34]
**16**	Linalyl acetate	*C. stellata* (f)	[16]
**17**	Limonene	*C. arvensis* (ae) *C. officinalis* (ae,f,l) *C. stellata* (f)	[16,30,31,34]
**18**	*p*-Menth-1-en-9-ol	*C. arvensis* (ae)	[34]
**19**	*p*-Metha-2,4-diene	*C. officinalis* (f)	[35]
**20**	Menthone	*C. officinalis* (f)	[35]
**21**	β-Myrcene	*C. arvensis* (ae) *C. officinalis* (f)	[36]
**22**	*trans*-β-Ocymene	*C. officinalis* (ae,l)	[30,31]
**23**	*neo*-*allo*-Ocymene	*C. officinalis* (f)	[29]
**24**	*trans*-Ocymenone	*C. officinalis* (f)	[29]
**25**	α-Phellandrene	*C. officinalis* (ae)	[30]
**26**	β-Phellandrene	*C. arvensis* (ae)	[34]
**27**	α-Pinene	*C. arvensis* (ae) *C. officinalis* (ae,f,l)	[30,31,34]
**28**	α-Pinene epoxide	*C. officinalis* (f)	[29]
**29**	β-Pinene	*C. arvensis* (ae) *C. officinalis* (ae,f,l)	[30,31,34]
**30**	*trans*-Pinocarveol	*C. officinalis* (f)	[29]
**31**	*iso*-Piperitenone	*C. officinalis* (f)	[29]
**32**	*cis*-Piperitol	*C. arvensis* (ae)	[34]
**33**	Sabinene	*C. arvensis* (ae) *C. officinalis* (ae,f,l)	[30,31,34]
**34**	*cis*-Sabinene hydrate	*C. arvensis* (ae)	[34]
**35**	Sabinyl acetate	*C. officinalis* (ae)	[30]
**36**	*cis*-Sesquisabinene hydrate	*C. arvensis* (ae)	[34]
**37**	*cis*-Tagetone	*C. officinalis* (f)	[29]
**38**	α-Terpinene	*C. officinalis* (ae)	[30]
**39**	α-Terpinene-7-al	*C. arvensis* (ae)	[34]
**40**	γ-Terpinene	*C. arvensis* (ae) *C. officinalis* (ae,f,l)	[30,31,33]
**41**	α-Terpineol	*C. officinalis* (ae,l)	[30,31]
**42**	Terpinene-4-ol	*C. arvensis* (ae) *C. officinalis* (ae,f,l)	[30,31,34]
**43**	α-Terpinolene	*C. arvensis* (ae) *C. officinalis* (f)	[29,34]
**44**	α-Thujene	*C. arvensis* (ae) *C. officinalis* (ae,f,l)	[30,31,34]
Sesquiterpenes			
**45**	β-Acoradiene	*C. arvensis* (ae)	[33]
**46**	β-Acorenol	*C. officinalis* (f)	[37]
**47**	α-Agarofuran	*C. arvensis* (ae)	[34]
**48**	α-Amorphene	*C. officinalis* (f,l)	[31]
**49**	δ-Amorphene	*C. arvensis* (ae)	[38]
**50**	Aromadendrene	*C. arvensis* (ae) *C. officinalis* (ae,f,l)	[30,31,34,37]
**51**	*allo*-Aromadendrene	*C. arvensis* (ae) *C. officinalis* (ae,f,l)	[30,31,34]
**52**	α-Bergamotene	*C. arvensis* (ae)	[34]
**53**	Bicyclogermacrene	*C. arvensis* (ae) *C. officinalis* (ae,f,l)	[34,36]
**54**	*epi*-Bicyclosesquiphellandrene	*C. officinalis* (ae,l)	[30,31]
**55**	α-Bisabolene	*C. arvensis* (ae)	[33]
**56**	β-Bisabolene	*C. arvensis* (ae)	[34]
**57**	α-Bisabolol	*C. arvensis* (ae) *C. officinalis* (f)	[34,37]
**58**	α-Bourbonene	*C. officinalis* (ae,l)	[30,31]
**59**	β-Bourbonene	*C. arvensis* (ae) *C. officinalis* (ae)	[34,36]
**60**	Bulnesol	*C. officinalis* (f)	[37]
**61**	cadalene	*C. officinalis* (f)	[32]
**62**	α-Cadinene	*C. arvensis* (ae) *C. officinalis* (ae,f,l)	[30,31,34,39]
**63**	γ-Cadinene	*C. arvensis* (ae) *C. officinalis* (ae,f,l)	[30,31,39]
**64**	δ-Cadinene	*C. arvensis* (ae) *C. officinalis* (ae,f,l)	[31,34,36]
**65**	Cadina-1,4-diene	*C. officinalis* (f,l)	[31,39]
**66**	*trans*-Cadina-1(6),4-diene	*C. officinalis* (ae)	[36]
**67**	*cis*-Cadina-1,4-diene	*C. arvensis* (ae) *C. officinalis* (ae)	[34,36]
**68**	*trans*-Cadina-1,4-diene	*C. arvensis* (ae) *C. officinalis* (ae)	[34,36]
**69**	Cadin-4-en-7-ol	*C. arvensis* (ae)	[34]
**70**	α-Cadinol	*C. arvensis* (ae) *C. officinalis* (ae)	[30,34]
**71**	τ-Cadinol	*C. arvensis* (ae) *C. officinalis* (ae,f)	[34,36,39]
**72**	α-Calacorene	*C. arvensis* (ae) *C. officinalis* (f)	[34,39]
**73**	β-Calacorene	*C. arvensis* (ae)	[34]
**74**	γ-Calacorene	*C. officinalis* (f,l)	[40]
**75**	*cis*-Calamene	*C. arvensis* (ae)	[34]
**76**	*trans*-Calamene	*C. arvensis* (ae)	[34]
**77**	Calamenene	*C. officinalis* (f,l)	[40]
**78**	Calarene	*C. officinalis* (ae,l)	[30,31]
**79**	Carota-3,8-diene	*C. arvensis* (ae)	[34]
**80**	Carotol	*C. officinalis* (f)	[35]
**81**	Caryophylla-2(12),6(13)-dien-5-one	*C. officinalis* (f)	[40]
**82**	α-Caryophyllene	*C. officinalis* (ae)	[36]
**83**	β-Caryophyllene	*C. arvensis* (ae) *C. officinalis* (ae,f,l)	[30,31,34,39]
**84**	Caryophyllene oxide	*C. arvensis* (ae) *C. officinalis* (ae)	[34,36]
**85**	Cedryl methyl ketone	*C. arvensis* (ae)	[34]
**86**	8,14-Cedranoxide	*C. arvensis* (ae) *C. officinalis* (ae) *C. suffruticosa* (ae)	[41]
**87**	α-Copaene	*C. arvensis* (ae) *C. officinalis* (ae,f,l)	[30,31,34,39]
**88**	β-Copaene	I (AE)	[36]
**89**	Copaene-4-ol	*C. officinalis* (f)	[32]
**90**	Cubebane-11-ol	*C. arvensis* (ae)	[34]
**91**	α-Cubebene	*C. arvensis* (ae) *C. officinalis* (ae,f,l)	[30,31,34,39]
**92**	β-Cubebene	*C. arvensis* (ae) *C. officinalis* (ae,f,l)	[30,31,34]
**93**	Cubebol	*C. arvensis* (ae) *C. officinalis* (f,l)	[34,40]
**94**	*epi*-Cubebol	*C. arvensis* (ae) *C. officinalis* (f,l)	[34,40]
**95**	Cubenol	*C. arvensis* (ae) *C. officinalis* (f)	[34,39]
**96**	*epi*-1-Cubenol	*C. arvensis* (ae) *C. officinalis* (ae)	[34,36]
**97**	*epi*-1,10-Dicubenol	*C. arvensis* (ae) *C. officinalis* (ae,f,l)	[32,34,36]
**98**	α-Curcumene	*C. arvensis* (ae)	[34]
**99**	β-Curcumene	*C. arvensis* (ae)	[33]
**100**	γ-Curcumene	*C. arvensis* (ae)	[33]
**101**	Elemene	*C. officinalis* (f)	[35]
**102**	β-Endobourbonene	*C. officinalis* (ae,f,l)	[30,31]
**103**	Epizonaren	*C. suffruticosa* (ae)	[42]
**104**	Eremoligenol	*C. arvensis* (ae)	[33]
**105**	Eremophylla-1(10),7-diene	*C. arvensis* (ae)	[34]
**106**	4β-5H-α-Eremophil-1(10)-en	*C. suffruticosa* (ae)	[42]
**107**	7-*epi*-α-Eudesmol	*C. officinalis* (f)	[35]
**108**	β-Eudesmol	*C. officinalis* (f)	[39]
**109**	γ-Eudesmol	*C. suffruticosa* (ae)	[42]
**110**	10-*epi*-γ-Eudesmol	*C. arvensis* (ae)	[34]
**111**	α-Farnesene	*C. arvensis* (ae)	[33]
**112**	β-Farnesene	*C. arvensis* (ae) *C. officinalis* (f)	[33,37]
**113**	(*E*, *Z*)-Farnesol	*C. arvensis* (ae)	[34]
**114**	(*Z*, *Z*)-Farnesol	*C. arvensis* (ae)	[34]
**115**	Germacradiene-11-ol	*C. arvensis* (ae)	[34]
**116**	Germacrene D	*C. arvensis* (ae) *C. officinalis* (ae,f,l)	[31,34,36,39]
**117**	Germacrene D-4-ol	*C. arvensis* (ae)	[34]
**118**	Gleenol	*C. arvensis* (ae)	[34]
**119**	Globulol	*C. arvensis* (ae)	[34]
**120**	*epi*-Globulol	*C. arvensis* (ae)	[33]
**121**	Guaiol	*C. arvensis* (ae) *C. officinalis* (f)	[34,37]
**122**	*cis*-β-Guaiene	*C. officinalis* (f) *C. suffruticosa* (ae)	[35,41]
**123**	α- Gurjunene	*C. arvensis* (ae) *C. officinalis* (ae,f,l)	[30,31,34,39]
**124**	β-Gurjunene	*C. officinalis* (f)	[39]
**125**	γ-Gurjunene	*C. officinalis* (f)	[37]
**126**	α-Himachalene	*C. officinalis* (f,l)	[40]
**127**	γ-Himachalene	*C. arvensis* (ae) *C. officinalis* (f,l)	[33,40]
**128**	α-Humulene	*C. arvensis* (ae) *C. officinalis* (ae,f,l)	[30,31,34,39]
**129**	γ-Humulene	*C. arvensis* (ae)	[34]
**130**	Isochiapin D	*C. suffruticosa* (ae)	[42]
**131**	Isocedranol	*C. officinalis* (f)	[37]
**132**	Isoledene	*C. arvensis* (ae)	[34]
**133**	α-Ionone	*C. officinalis* (f)	[39]
**134**	β-Ionone	*C. officinalis* (f)	[39]
**135**	Ledane	*C. officinalis* (f)	[39]
**136**	Ledene	*C. arvensis* (ae) *C. officinalis* (f) *C. suffruticosa* (ae)	[34,39,42]
**138**	Ledol	*C. arvensis* (ae) *C. officinalis* (f)	[34,39]
**139**	Longifolene	*C. officinalis* (f)	[35]
**140**	Longipinene	*C. arvensis* (ae)	[33]
**141**	α-Muurolene	*C. arvensis* (ae) *C. officinalis* (ae,f,l)	[30,31,34,39]
**142**	γ-Muurolene	*C. arvensis* (ae) *C. officinalis* (ae,f)	[34,36,39]
**143**	*epi*-α-Muurolol	*C. officinalis* (f)	[32]
**144**	τ-Muurolol	*C. arvensis* (ae) *C. officinalis* (f,l)	[31,34,39]
**145**	*cis*-Muurola-3,5-diene	*C. arvensis* (ae) *C. officinalis* (ae)	[34,36]
**146**	*trans*-Muurola-3,5-diene	*C. arvensis* (ae)	[36]
**147**	*cis*-Muurola-4(14),5-diene	*C. arvensis* (ae) *C. officinalis* (ae)	[34,36]
**148**	Muurol-5-en-4-B-ol	*C. officinalis* (f)	[37]
**149**	Nerolidol	*C. arvensis* (ae) *C. officinalis* (ae,f)	[30,31,34]
**150**	β-Oplopenone	*C. arvensis* (ae) *C. officinalis* (ae,l)	[30,31,34]
**151**	α-Oxobisabolene	*C. arvensis* (ae)	[33]
**152**	Palustrol	*C. arvensis* (ae) *C. officinalis* (ae,l)	[30,31,34]
**153**	α-Patchoulene	*C. officinalis* (f)	[37]
**154**	α-Patchouli alcohol	*C. officinalis* (f)	[37]
**155**	β-Patchouli alcohol	*C. officinalis* (f)	[37]
**156**	Presilphiperfolane-9α-ol	*C. arvensis* (ae)	[34]
**157**	α-Santalol	*C. arvensis* (ae)	[38]
**158**	α-Selinene	*C. arvensis* (ae)	[38]
**159**	β-Selinene	*C. officinalis* (ae,l)	[30,31]
**160**	γ-Selinene	*C. officinalis* (ae) *C. suffruticosa* (ae)	[36,42]
**161**	*Z*-Sesquilavandulol	*C. arvensis* (ae)	[38]
**162**	β-Sesquiphellandrene	*C. arvensis* (ae)	[33]
**163**	7-β-Silphiperfol-5-ene	*C. arvensis* (ae) *C. suffruticosa* (ae)	[38,42]
**164**	Spatulenol	*C. arvensis* (ae) *C. officinalis* (f)	[29,34]
**165**	Valerianol	*C. officinalis* (ae)	[41]
**166**	Valencene	*C. arvensis* (ae)	[34]
**167**	Verbenol	*C. officinalis* (f)	[29]
**168**	Viridiflorene	*C. arvensis* (ae)	[38]
**169**	Viridiflorol	*C. arvensis* (ae) *C. officinalis* (f)	[32,33]
**170**	α-Ilangene	*C. officinalis* (ae,l)	[30,31]
**171**	Zingiberene	*C. arvensis* (ae)	[33]
**172**	Zingiberenol	*C. arvensis* (ae)	[34]
**173**	Zonarene	*C. arvensis* (ae)	[34]
	Sesquiterpene glycosides		
**174**	4-*epi*-Cubebol *O*-^βD^Fuc*p* (arvoside A)	*C. arvensis* (ae)	[43]
**175**	Viridiflorol *O*-^βD^Fuc*p* (arvoside B)	*C. arvensis* (ae)	[44]
**176**	Viridiflorol *O*-^βD^Fuc*p* 2′-*O*-acetate	*C. arvensis* (ae)	[44]
**177**	Viridiflorol *O*-^βD^Fuc*p* 2′-*O*-isobutyrate	*C. officinalis* (f)	[45]
**178**	Viridiflorol *O*-^βD^Fuc*p* 2′-*O*-isovalerate	*C. arvensis* (ae)	[44]
**179**	Viridiflorol *O*-^βD^Fuc*p* 2′-*O*-methylpentenoate	*C. arvensis* (ae)	[44,46]
**180**	Viridiflorol *O*-^βD^Fuc*p* 2′-*O*-methylpropanoate	*C. arvensis* (ae)	[47,48]
**181**	Viridiflorol *O*-^βD^Fuc*p* 2′-*O*-methylbutenoate	*C. arvensis* (ae)	[46,47]
**182**	Viridiflorol *O*-^βD^Fuc*p* 2′-*O*-senecioate	*C. officinalis* (ae,f)	[45,46]
**183**	Viridiflorol *O*-^βD^Fuc*p* 2′-*O*-(4-methylsenecioate)	*C. arvensis* (ae)	[46]
**184**	Viridiflorol *O*-^βD^Fuc*p* 2′-*O*-angelate	*C. officinalis* (f)	[45]
**185**	Viridiflorol *O*-^βD^Fuc*p* 2′-*O*-tiglate	*C. officinalis* (f)	[45]
**186**	Viridiflorol *O*-^βD^Fuc*p* 2′-*O*-(3-methyl-2-pentenoate)	*C. officinalis* (f)	[45]
**187**	Viridiflorol *O*-^βD^Chi*p*	*C. arvensis* (ae)	[46]
**188**	Viridiflorol *O*-^βD^Chi*p* 2′-*O*-senecioate	*C. arvensis* (ae)	[46]
**189**	Viridiflorol *O*-^βD^Chi*p* 2′-*O*-(3-methyl-2-pentenoate)	*C. officinalis* (f)	[49]
**190**	β-Eudesmol *O*-^βD^Fuc*p* 2′-*O*-angelate	*C. officinalis* (ae,f)	[45,46]
**191**	β-Eudesmol *O*-^βD^Fuc*p* 2′-*O*-tiglate	*C. officinalis* (f)	[45]
**192**	β-Eudesmol *O*-^βD^Fuc*p* 2′-*O*-senecioate	*C. officinalis* (f)	[45]
**193**	β-Eudesmol *O*-^βD^Fuc*p* 2′-*O*-isobutyrate	*C. officinalis* (f)	[45]
**194**	β-Eudesmol *O*-^βD^Fuc*p* 2′-*O*-(2-methylbutyrate)	*C. officinalis* (f)	[45]
**195**	β-Eudesmol *O*-^βD^Fuc*p* 2′-*O*-(3-methyl-2-pentenoate)	*C. officinalis* (f)	[45]
**196**	β-Eudesmol *O*-^βD^Chi*p* 2′-*O*-angelate157	*C. arvensis* (ae)	[46]
**197**	4α-Hydroxygermacra-1(10)*E*,5*E*-diene *O*-^βD^Fuc*p* 2′-*O*-angelate	*C. arvensis* (ae)	[46]
**198**	3,7,11-Trimethy1-1,6-dodecadien-3,10,11-triol 3-*O*-^βD^Glc*p* (icariside C_3_)	*C. officinalis* (f)	[9]
**199**	(3*S*,5*R*,8*S*,9ζ)-5,8-Epoxy-6-megastigmene-3,9-diol 3-*O*-^βD^Glc*p* (officinoside A)	*C. officinalis* (f)	[50]
**200**	(3*S*,5*R*,8*R*,9*R*)-5,8-Epoxy-6-megastigmene-3,9-diol 3-*O*-^βD^Glc*p* (officinoside B)	*C. officinalis* (f)	[50]
**201**	Selin-4(15)-ene-3β,11-diol 3-*O*-^βD^Glc*p*-12-*O*-^βD^Fuc*p* (officinoside C)	*C. officinalis* (f)	[50]
**202**	Flourensadiol 10-*O*-^βD^Glc*p*-12-*O*-^βD^Fuc*p* (officinoside D)	*C. officinalis* (f)	[50]
**203**	3α,7β-Dihydroxy-5β,6β-epoxyeudesm-4(15)-ene 11-*O*-^βD^Fuc*p* 2′,4′-di-*O*-angelate-3′-*O*-acetate	*C. arvensis* (ae)	[48]
**204**	3α,7β-Dihydroxy-5β,6β-epoxyeudesm-4(15)-ene 11-*O*-^βD^Fuc*p* 2′,4′-di-*O*-angelate-3′-*O*-isobutyrate	*C. arvensis* (ae)	[48]
**205**	3α,7β-Dihydroxy-5β,6β-epoxyeudesm-4(15)-ene 11-*O*-^βD^Fuc*p* 2′,4′-di-*O*-angelate-3′-*O*-methylbutyrate	*C. arvensis* (ae)	[48]
**206**	3α,7β-Dihydroxy-15-acetoxyeudesm-4(5)-ene 11-*O*-^βD^Fuc*p* 2′,4′-di-*O*-angelate-3′-*O*-acetate	*C. arvensis* (ae)	[48]
**207**	α-Elemol *O*-^βD^Fuc*p* 2′-*O*-angelate	*C. officinalis* (f)	[45]
	Diterpenes		
**208**	Neophytadiene	*C. arvensis* (ae) *C. officinalis* (ae,f,l) *C. suffruticosa* (ae)	[40,41]
**209**	Phytol	*C. arvensis* (ae) *C. officinalis* (ae) *C. suffruticosa* (ae)	[34,41]
	Triterpenes: aliphatic		
**210**	Squalene	*C. suffruticosa* (ae)	[42]
	Triterpenes: stigmastane derivatives		
**211**	Stigmastane-5-ene	*C. arvensis* (ae) *C. officinalis* (ae) *C. suffruticosa* (ae)	[41]
**212**	Stigmastane-3β-ol (stigmastanol)	*C. officinalis* (l)	[51]
**213**	Stigmast-5-en-3β-ol (β-sitosterol)	*C. officinalis* (f,l,r,s)	[51,52,53,54]
**214**	Stigmast-7-en-3β-ol (Δ-7-sitosterol)	*C. officinalis* (l,s)	[51,52]
**215**	Stigmasta-5,22-dien-3β-ol (stigmasterol)	*C. arvensis* (ae) *C. officinalis* (f,l,r,s) *C. suffruticosa* (ae)	[41,51,52,53,54]
**216**	Stigmasta-5,24(28)-dien-3β-ol (Δ-5-avenasterol, isofucosterol)	*C. officinalis* (f,l,r,s)	[52,53,54]
**217**	Stigmasta-5,25-dien-3β-ol (clerosterol)	*C. officinalis* (l)	[51]
**218**	Stigmasta-7,24(28)-dien-3β-ol (Δ-7-avenasterol)	*C. officinalis* (s)	[52]
**219**	Stigmasta-7,24-dien-3β-ol 4-methyl ester (citrostadienol)	*C. officinalis* (s)	[52]
**220**	Stigmasta-3,6-dione	*C. officinalis* (l)	[54]
	Triterpenes: ergostane derivatives		
**221**	Ergostan-3β-ol (campestanol)	*C. officinalis* (l,s)	[51,52]
**222**	Ergost-5-en-3β-ol (campesterol)	*C. officinalis* (f,l,r,s)	[51,52,53,54]
**223**	Ergost-7-en-3β-ol (Δ-7-campesterol)	*C. officinalis* (s)	[52]
**224**	Ergosta-5,22-dien-3β-ol (brassicasterol)	*C. officinalis* (l)	[51]
	Triterpenes: cholestane derivatives		
**225**	Cholestan-3β-ol	*C. officinalis* (l)	[51]
**226**	Cholest-5-en-3β-ol (cholesterol)	*C. officinalis* (l,s)	[51,52]
**227**	24-Methylen-cholesterol	*C. officinalis* (l)	[51]
**228**	Cholest-7-en-3β-ol	*C. officinalis* (l)	[51]
**229**	4*β*-Metylcholest-20-en-12-ol-3*β*-olide (calendulosterolide)	*C. officinalis* (f)	[55]
	Triterpenes: lanostane derivatives		
**230**	Lanost-20(22)-en-3β-ol	*C. officinalis* (f)	[55]
**231**	Lanosta-8,24-dien-3β-ol (lanosterol)	*C. suffruticosa* (ae)	[42]
	Triterpenes: dammarane derivatives		
**232**	Dammara-20,24-dien-3β-ol (dammaradienol)	*C. officinalis* (f)	[3]
	Triterpenes: cycloartane derivatives		
**233**	9,19-Cyclolanost-24-en-3β-ol (cycloartenol)	*C. officinalis* (f)	[3]
**234**	24-Methylenecycloartanol	*C. officinalis* (f,l,r)	[3,54]
	Triterpenes: friedelane derivatives		
**235**	Friedelane-3β-ol (friedelanol)	*C. officinalis* (r)	[54]
**236**	Friedelane-3-one (friedelin)	*C. officinalis* (r)	[54]
	Triterpenes: lupane derivatives		
**237**	Lupane-3β,16β,20-triol	*C. officinalis* (f)	[56]
**238**	Lupane-3β,16β,20-triol 3-*O*-myristate	*C. officinalis* (f)	[56]
**239**	Lupane-3β,16β,20-triol 3-*O*-palmitate	*C. officinalis* (f)	[56]
**240**	Lup-20(29)-en-3β-ol (lupeol)	*C. officinalis* (f,l,r,s) *C. suffruticosa* (ae)	[41,53,54,57]
**241**	Lup-20(29)-ene-3β,16β-diol (calenduladiol)	*C. officinalis* (f)	[53,57]
**242**	Calenduladiol 3-*O*-laurate	*C. officinalis* (f)	[58]
**243**	Calenduladiol 3-*O*-myristate	*C. officinalis* (f)	[58]
**244**	Calenduladiol 3-*O*-palmitate	*C. officinalis* (f)	[58]
**245**	Lup-20(29)-eh-3β,16β,28-triol	*C. officinalis* (f)	[59]
**246**	Lup-20(29)-en-28-al	*C. arvensis* (ae) *C. officinalis* (ae) *C. suffruticosa* (ae)	[41]
	Triterpenes: ursane derivatives		
**247**	Urs-12-en-3β-ol (α-amyrin)	*C. officinalis* (f,l,r,s) *C. suffruticosa* (ae)	[42,53,54,57]
**248**	α-Amyrin 3-*O*-laurate	*C. officinalis* (f)	[53]
**249**	α-Amyrin 3-*O*-myristate	*C. officinalis* (f)	[53]
**250**	α-Amyrin 3-*O*-palmitate	*C. officinalis* (f)	[53]
**251**	Urs-20(30)-en-3β-ol (taraxasterol)	*C. officinalis* (f)	[53,54]
**252**	Urs-20-en-3β-ol (ψ-taraxasterol)	*C. officinalis* (f)	[53,54,57]
**253**	Urs-20-ene-3β,12β-diol (faradiol)	*C. officinalis* (f)	[53,57]
**254**	Faradiol 3-*O*-laurate	*C. officinalis* (f)	[60]
**255**	Faradiol 3-*O*-myristate	*C. officinalis* (f)	[60]
**256**	Faradiol 3-*O*-palmitate	*C. officinalis* (f)	[60]
**257**	Urs-20(30)-ene-3β,16β-diol (arnidiol)	*C. officinalis* (f)	[53,57]
**258**	Arnidiol 3-*O*-laurate	*C. officinalis* (f)	[58]
**259**	Arnidiol 3-*O*-myristate	*C. officinalis* (f)	[58]
**260**	Arnidiol 3-*O*-palmitate	*C. officinalis* (f)	[58]
**261**	3-Hydroxyurs-2,20-dien-28-al	*C. officinalis* (ae) *C. suffruticosa* (ae)	[41]
**262**	3-Oxoursan-28-oic acid	*C. arvensis* (ae) *C. officinalis* (ae) *C. suffruticosa* (ae)	[41]
**263**	Brein 205	*C. officinalis* (f)	[53,57]
**264**	Ursa-12-ene-3*β*,16*β*,21-triol	*C. officinalis* (f)	[59]
**265**	Urs-12-en-3-on (α-amyrenone)	*C. officinalis* (f)	[54]
**266**	Tarax-20-en-3*β*,16*β*,21*α*-triol (arnitriol A)	*C. officinalis* (f)	[56]
**267**	Arnitriol A 3-*O*-myristate	*C. officinalis* (f)	[56]
**268**	Arnitriol A 3-*O*-palmitate	*C. officinalis* (f)	[56]
**269**	Tarax-20-en-3*β*,16*β*,22*α*-triol (heliantriol C)	*C. officinalis* (f)	[59]
**270**	Tarax-20-en-3*β*,16*β*,30-triol (heliantriol F)	*C. officinalis* (f)	[59]
	Triterpenes: oleanane derivatives		
**271**	Olean-12-en-3β-ol (β-amyrin)	*C. officinalis* (f,l,r,s) *C. suffruticosa* (ae)	[42,52,53,54,57]
**272**	β-Amyrin 3-*O*-laurate	*C. officinalis* (f)	[53]
**273**	β-Amyrin 3-*O*-myristate	*C. officinalis* (f)	[53]
**274**	β-Amyrin 3-*O*-palmitate	*C. officinalis* (f)	[53]
**275**	β-Amyrin acetate	*C. officinalis* (f)	[61]
**276**	Olean-13(18)-ene-3β,16β-diol (ursadiol)	*C. officinalis* (f)	[53,62]
**277**	Ursadiol 3-*O*-laurate	*C. officinalis* (f)	[53]
**278**	Ursadiol 3-*O*-myristate	*C. officinalis* (f)	[53]
**279**	Ursadiol 3-*O*-palmitate	*C. officinalis* (f)	[53]
**280**	Olean-13(18)-ene-3β,16β-dion (ursadione)	*C. officinalis* (f)	[63]
**281**	Olean-12-ene-3β,16β-diol (maniladiol)	*C. officinalis* (f)	[60]
**282**	Maniladiol 3-*O*-myristate	*C. officinalis* (f)	[60]
**283**	Maniladiol 3-*O*-palmitate	*C. officinalis* (f)	[60]
**284**	Olean-12-ene-3,28-diol (erythrodiol)	*C. officinalis* (f)	[53,64]
**285**	Oleane-12-en-3β,16β,28-triol	*C. officinalis* (f)	[59]
**286**	3β-Hydroxyolean-12-en-28-oic acid (oleanolic acid)	*C. officinalis* (ae,r)	[57]
**287**	Oleanolic acid methyl ester	*C. officinalis* (f,r)	[54]
**288**	Oleanolic acid methyl ester 3-*O*-acetate	*C. officinalis* (r)	[54]
**289**	Oleanolic acid 3-*O*-acetate	*C. officinalis* (ae)	[65]
**290**	Oleanolic acid 3-*O*-acetate methyl ester	*C. officinalis* (r)	[66]
**291**	Oleanolic acid 3-*O*-^βD^GlcA*p* (glucoside F, glucuronide F, calenduloside E, calendulaglycoside F, momordin Ib, polysciasaponin P_7_, silphioside F)	*C. officinalis* (ae,r)	[9,67,68,69,70]
**292**	Oleanolic acid 3-*O*-(6′-*O*-Me)-^βD^GlcA*p* (glucoside F methyl ester)	*C. officinalis* (ae,r)	[71]
**293**	Oleanolic acid 3-*O*-(2′-*O*-^βD^Glc*p*)-^βD^GlcA*p* (glucoside E, zingibroside R_1_, ginsenoside Z-R1, polysciasaponin P5, deglucosylchikusetsusaponin V)	*C. officinalis* (ae) *C. stellata* (w)	[67,72]
**294**	Oleanolic acid 3-*O*-(2′-*O*-^βD^Gal*p*)-^βD^GlcA*p* (udosaponin B)	*C. stellata* (w)	[72]
**295**	Oleanolic acid 3-*O*-(3′-*O*-^βD^Gal*p*)-^βD^GlcA*p* (glucoside D, glucuronide D, calenduloside G, calendulaglycoside G)	*C. arvensis* (ae) *C. officinalis* (ae,r) *C. stellata* (w) *C. suffruticosa* (ae)	[9,67,68,70,72,73,74,75]
**296**	Oleanolic acid 3-*O*-(2′-*O*-^βD^Glc*p*-3′-*O*-^βD^Gal*p*)-^βD^GlcA*p* (glucoside B, glucuronide B, calendulaglycoside B)	*C. officinalis* (ae)	[67,68,70]
**297**	Oleanolic acid 3-*O*-(3′-*O*-^βD^Gal*p*-6′-*O*-Me)-^βD^GlcA*p* (calenduloside G methyl ester)	*C. officinalis* (f)	[7]
**298**	Oleanolic acid 3-*O*-(2′-*O*-^βD^Glc*p*-3′-*O*-^βD^Gal*p*-6′-*O*-But)-^βD^GlcA*p* (glucoside B butyl ester, calendulaglycoside B butyl ester)	*C. officinalis* (f)	[7]
**299**	Oleanolic acid 3-*O*-^βD^GlcA*p*-28-*O*-^βD^Glc*p* (glucoside D_2_, glucuronide D_2_, calenduloside F, momordin IIb, silphioside G, chikusetsusaponin IVa)	*C. officinalis* (ae,r) *C. stellata* (w)	[9,68,70,72,76]
**300**	Oleanolic acid 3-*O*-(3′-*O*-^βD^Gal*p*)-^βD^GlcA*p*-28-*O*-^βD^Glc*p* (glucoside C, glucuronide C, calendulaglycoside C, calenduloside H)	*C. arvensis* (ae) *C. officinalis* (ae,r) *C. stellata* (w)	[67,68,70,72,73,74]
**301**	Oleanolic acid 3-*O*-(3′-*O*-dCrt)-^βD^GlcA*p*-28-*O*-^βD^Glc*p* (arvensoside C)	*C. arvensis* (ae)	[77]
**302**	Oleanolic acid 3-*O*-(6′-*O*-Me)-^βD^GlcA*p*-28-*O*-^βD^Glc*p* (glucoside D_2_ butyl ester, calenduloside F butyl ester)	*C. officinalis* (f)	[7]
**303**	Oleanolic acid 3-*O*-(2′-*O*-^βD^Glc*p*-3′-*O*-^βD^Gal*p*)-^βD^GlcA*p*-28-*O*-^βD^Glc*p* (glucoside A, glucuronide A, calendulaglycoside A)	*C. officinalis* (ae) *C. stellata* (w)	[67,68,70,72]
**304**	Oleanolic acid 3-*O*-(2′-*O*-^βD^Glc*p*-3′-*O*-^βD^Gal*p*-6′-*O*-Me)-^βD^GlcA*p*-28-*O*-^βD^Glc*p* (glucoside A methyl ester, calendulaglycoside A methyl ester)	*C. officinalis* (f)	[7]
**305**	Oleanolic acid 3-*O*-(3′-*O*-^βD^Gal*p*-6′-*O*-Me)-^βD^GlcA*p*-28-*O*-^βD^Glc*p* (glucoside C methyl ester, calendulaglycoside C methyl ester)	*C. officinalis* (f)	[7]
**306**	Oleanolic acid 3-*O*-(3′-*O*-^βD^Gal*p*-6′-*O*-But)-^βD^GlcA*p*-28-*O*-^βD^Glc*p* (calendulaglycoside C butyl ester)	*C. officinalis* (f)	[7]
**307**	Oleanolic acid 3-*O*-(2′-*O*-^βD^Glc*p*-3′-*O*-^βD^Gal*p*-6′-*O*-But)-^βD^GlcA*p*-28-*O*-^βD^Glc*p* (calendulaglycoside A butyl ester)	*C. officinalis* (f)	[7]
**308**	Oleanolic acid 3-*O*-^βD^Glc*p* (glucoside I)	*C. officinalis* (ae,r)	[71]
**309**	Oleanolic acid 3-*O*-(3′-*O*-^βD^Gal*p*)-^βD^Glc*p* (arvensoside B)	*C. arvensis* (ae) *C. stellata* (w)	[72,73,78]
**310**	Oleanolic acid 3-*O*-(4′-*O*-^βD^Gal*p*)-^βD^Glc*p* (glucoside II, calenduloside A)	*C. officinalis* (ae,r)	[68,71,79]
**311**	Oleanolic acid 3-*O*-(4′-*O*-(4″-*O*-^βD^Gal*p*)-^βD^Gal*p*)-^βD^Glc*p* (glucoside III)	*C. officinalis* (ae,r)	[71]
**312**	Oleanolic acid 3-*O*-(2′-*O*-^βD^Glc*p*-3′-*O*-^βD^Gal*p*)-^βD^Glc*p* (calenduloside C, osteosaponin-I, elateroside B, 2′′,28-dideglucosylosteosaponin II)	*C. arvensis* (ae) *C. officinalis* (r) *C. stellata* (w)	[72,80]
**313**	Oleanolic acid 3-*O*-(3′-*O*-^βD^Glc*p*-4′-*O*-^βD^Gal*p*)-^βD^Glc*p* (glucoside IV)	*C. officinalis* (ae,r)	[71]
**314**	Oleanolic acid 3-*O*-(3′-*O*-(3″-*O*-^βD^Glc*p*)-^βD^Glc*p*-4′-*O*-^βD^Gal*p*)-^βD^Glc*p* (glucoside V)	*C. officinalis* (ae,r)	[71]
**315**	Oleanolic acid 3-*O*-(3′-*O*-(3″-*O*-^βD^Glc*p*)-^βD^Glc*p*-4′-*O*-(4‴-*O*-^βD^Gal*p*)-^βD^Gal*p*)-^βD^Glc*p* (glucoside VI)	*C. officinalis* (ae,r)	[71]
**316**	Oleanolic acid 3-*O*-(3′-*O*-(3″-*O*-(3″-*O*-^βD^Glc*p*)-^βD^Glc*p*)-^βD^Glc*p*-4′-*O*-^βD^Gal*p*)-^βD^Glc*p* (glucoside VII)	*C. officinalis* (ae,r)	[71]
**317**	Oleanolic acid 3,28-*O*-^βD^Glc*p*_2_ (silphioside B)	*C. stellata* (w)	[72]
**318**	Oleanolic acid 3-*O*-(3′-*O*-^βD^Gal*p*)-^βD^Glc*p*-28-*O*-^βD^Glc*p* (arvensoside A)	*C. arvensis* (ae) *C. officinalis* (f)	[78]
**319**	Oleanolic acid 3-*O*-(4′-*O*-^βD^Gal*p*)-^βD^Glc*p*-28-*O*-^βD^Glc*p* (calenduloside B)	*C. officinalis* (r) *C. stellata* (w)	[72,81]
**320**	Oleanolic acid 3-*O*-(2′-*O*-^βD^Glc*p*-3′-*O*-^βD^Gal*p*)-^βD^Glc*p*-28-*O*-^βD^Glc*p* (calenduloside D)	*C. arvensis* (ae) *C. officinalis* (f,r) *C. stellata* (w)	[72,80]
**321**	Oleanolic acid 3-*O*-(3′-*O*-(3″-*O*-^βD^Glc*p*)-^βD^Glc*p*-4′-*O*-^βD^Gal*p*)-^βD^Glc*p*-28-*O*-^βD^Glc*p* (glucoside VIII)	*C. officinalis* (ae,r)	[71]
**322**	Oleanolic acid 3-*O*-(3′-*O*-^βD^Gal*p*-4′-*O*-^βD^Glc*p*)-^βD^Glc*p*-28-*O*-^βD^Glc*p*	*C. arvensis* (ae)	[82]
**323**	3β-Hydroxyolean-18-en-28-oic acid (morolic acid)	*C. stellata* (w)	[72]
**324**	Morolic acid 3-*O*-(2′-*O*-^βD^Glc*p*-3′-*O*-^βD^Gal*p*)-^βD^Glc*p* (calendustellatoside D)	*C. stellata* (w)	[72]
**325**	3-Oxoolean-18-en-28-oic acid (moronic acid)	*C. officinalis* (f)	[9]
**326**	Moronic acid 3-*O*-(2′-*O*-^βD^Glc*p*-3′-*O*-^βD^Gal*p*)-^βD^GlcA*p*-28-*O*-^βD^Glc*p* (calendasaponin A)	*C. officinalis* (f)	[9]
**327**	3β,16α-Dihydroxyolean-12-en-28-oic acid (echinocystic acid)	*C. stellata* (w)	[72]
**328**	Echinocystic acid 3-*O*-^βD^GlcA*p*-28-*O*-^βD^Glc*p* (acanthopanaxoside E)	*C. stellata* (w)	[72]
**329**	Echinocystic acid 3-*O*-(3′-*O*-^βD^Gal*p*)-^βD^Glc*p*-28-*O*-^βD^Glc*p* (calendustellatoside B)	*C. stellata* (w)	[72]
**330**	Echinocystic acid 3-*O*-(3′-*O*-(6‴-O-Mal)-^βD^Gal*p*)-^βD^Glc*p*-28-*O*-^βD^Glc*p* (calendustellatoside C)	*C. stellata* (w)	[72]
**331**	Echinocystic acid 3-*O*-(2′-*O*-^βD^Glc*p*-3′-*O*-^βD^Gal*p*)-^βD^Glc*p*-28-*O*-^βD^Glc*p* (calendustellatoside A)	*C. stellata* (w)	[72]
**332**	3β,16β-Dihydroxyolean-12-en-28-oic acid (cochalic acid)	*C. officinalis* (f)	[9]
**333**	Cochalic acid 3-*O*-(3′-*O*-^βD^Gal*p*)-^βD^GlcA*p*-28-*O*-^βD^Glc*p* (calendasaponin B)	*C. officinalis* (f) *C. suffruticosa* (ae)	[9,75]
**334**	Cochalic acid 3-*O*-(2′-*O*-^βD^Glc*p*-3′-*O*-^βD^Gal*p*)-^βD^GlcA*p*-28-*O*-^βD^Glc*p* (calendasaponin C)	*C. officinalis* (f)	[9]
**335**	3β,21β-Dihydroxyolean-12-en-28-oic acid (machaerinic acid)	*C. officinalis* (f)	[9]
**336**	Machaerinic acid 3-*O*-(2′-*O*-^βD^Gal*p*)-^βD^GlcA*p*-28-*O*-^βD^Glc*p*	*C. stellata* (w)	[72]
**337**	Machaerinic acid 3-*O*-(2′-*O*-^βD^Glc*p*-3′-*O*-^βD^Gal*p*)-^βD^GlcA*p*-28-*O*-^βD^Glc*p* (calendasaponin D)	*C. officinalis* (f)	[9]
**338**	3β,29-Dihydroxyolean-12-en-28-oic acid (mesembryanthemoidigenic acid)	*C. stellata* (w)	[72]
**339**	Mesembryanthemoidigenic acid 3-*O*-(3′-*O*-^βD^Gal*p*)-^βD^GlcA*p*-28-*O*-^βD^Glc*p* (calendustellatoside E)	*C. stellata* (w)	[72]
**340**	3β-Acetoxyoleane-12-en-27-oic acid (cornulacic acid)	*C. officinalis* (ae)	[65]
	Triterpenes: tirucallane derivatives		
**341**	Helianol	*C. officinalis* (f)	[3]
**342**	Tirucalla-7,24-dienol	*C. officinalis* (f)	[3]
	Carotenoids		
**343**	Antheraxanthin	*C. officinalis* (f)	[83]
**344**	(9*Z*)-Antheraxanthin	*C. officinalis* (f)	[84]
**345**	(all-*E*)-Antheraxanthin	*C. officinalis* (f)	[85]
**346**	Auroxanthin	*C. officinalis* (f)	[83]
**347**	Auroxanthin stearate	*C. officinalis* (f)	[85]
**348**	(all-*E*)-Auroxanthin	*C. officinalis* (f)	[85]
**349**	(all-*E*)-Auroxanthin palmitate	*C. officinalis* (f)	[85]
**350**	α-Carotene	*C. officinalis* (f)	[83]
**351**	β-Carotene	*C. officinalis* (f)	[83]
**352**	(13*Z*)-β-Carotene	*C. officinalis* (f)	[85]
**353**	(15*Z*)-β-Carotene	*C. officinalis* (f)	[85]
**354**	(all-*E*)-β-Carotene	*C. officinalis* (f)	[85]
**355**	(*Z*)-β-Carotene	*C. officinalis* (f)	[84]
**356**	γ-Carotene	*C. officinalis* (f)	[84]
**357**	γ-Carotene 1′,2′-epoxide	*C. officinalis* (f)	[85]
**358**	(5′*Z*)-γ-Carotene	*C. officinalis* (f)	[84]
**359**	δ-Carotene	*C. officinalis* (f)	[84]
**360**	δ-Carotene 1′,2′-epoxide	*C. officinalis* (f)	[85]
**361**	Chrysanthemaxanthin	*C. officinalis* (f)	[83]
**362**	α-Cryptoxanthin	*C. officinalis* (f)	[83]
**363**	β-Cryptoxanthin	*C. officinalis* (f)	[84]
**364**	β-Cryptoxanthin laurate	*C. officinalis* (f)	[85]
**365**	β-Cryptoxanthin palmitate	*C. officinalis* (f)	[85]
**366**	β-Cryptoxanthin stearate	*C. officinalis* (f)	[85]
**367**	(all-*E*)-β-Cryptoxanthin	*C. officinalis* (f)	[85]
**368**	(all-*E*)-β-Cryptoxanthin myristate	*C. officinalis* (f)	[85]
**369**	(*Z*)-Cryptoxanthin	*C. officinalis* (f)	[84]
**370**	Flavoxanthin	*C. officinalis* (f)	[83]
**371**	Lactucaxanthin	*C. officinalis* (f)	[86]
**372**	Lycopene	*C. officinalis* (f)	[83]
**373**	(5*Z*, 9*Z*)-Lycopene	*C. officinalis* (f)	[84]
**374**	(5*Z*, 9*Z*, 5′*Z*)-Lycopene	*C. officinalis* (f)	[84]
**375**	(5*Z*, 9*Z*, 5′*Z*, 9′*Z*)-Lycopene	*C. officinalis* (f)	[84]
**376**	(9/9′)-Lutein	*C. officinalis* (f)	[83]
**377**	(13/13′)-Lutein	*C. officinalis* (f)	[84]
**378**	(9*Z*)-Lutein	*C. officinalis* (f)	[84]
**379**	(13′*Z*)-Lutein	*C. officinalis* (f)	[85]
**380**	(all-*E*)-Lutein	*C. officinalis* (f)	[85]
**381**	(all-*E*)-Lutein 3-*O*-myristate	*C. officinalis* (f)	[85]
**382**	(all-*E*)-Lutein 3′-*O*-myristate	*C. officinalis* (f)	[85]
**383**	(all-*E*)-Lutein 3-*O*-palmitate	*C. officinalis* (f)	[85]
**384**	(all-*E*)-Lutein 3′-*O*-palmitate	*C. officinalis* (f)	[85]
**385**	(all-*E*)-Lutein 3-*O*-stearate	*C. officinalis* (f)	[85]
**386**	(all-*E*)-Lutein 3′-*O*-stearate	*C. officinalis* (f)	[85]
**387**	(all-*E*)-Lutein dilaurate	*C. officinalis* (f)	[85]
**388**	(all-*E*)-Lutein dimyristate	*C. officinalis* (f)	[85]
**389**	(all-*E*)-Lutein distearate	*C. officinalis* (f)	[85]
**390**	(all-*E*)-Lutein 3-*O*-laurate-3′-*O*-caprate	*C. officinalis* (f)	[85]
**391**	(all-*E*)-Lutein 3-*O*-laurate-3′-*O*-myristate	*C. officinalis* (f)	[85]
**392**	(all-*E*)-Lutein 3-*O*-myristate-3′-*O*-laurate	*C. officinalis* (f)	[85]
**393**	(all-*E*)-Lutein 3-*O*-myristate-3′-*O*-palmitate	*C. officinalis* (f)	[85]
**394**	(all-*E*)-Lutein 3-*O*-palmitate-3′-*O*-myristate	*C. officinalis* (f)	[85]
**395**	(all-*E*)-Lutein 3-*O*-myristate-3′-*O*-stearate	*C. officinalis* (f)	[85]
**396**	(all-*E*)-Lutein 3-*O*-stearate-3′-*O*-myristate	*C. officinalis* (f)	[85]
**397**	(all-*E*)-Lutein 3-*O*-palmitate-3′-*O*-stearate	*C. officinalis* (f)	[85]
**398**	(all-*E*)-Lutein 3-*O*-stearate-3′-*O*-palmitate	*C. officinalis* (f)	[85]
**399**	(*Z*)-Lutein dilaurate	*C. officinalis* (f)	[85]
**400**	(Z)-Lutein dimyristate	*C. officinalis* (f)	[85]
**401**	Lutein dibutyrate	*C. officinalis* (f)	[85]
**402**	Lutein dicaprylate	*C. officinalis* (f)	[85]
**403**	Lutein dicaprate	*C. officinalis* (f)	[85]
**404**	Lutein dipalmitate	*C. officinalis* (f)	[85]
**405**	Lutein-5,6-epoxide	*C. officinalis* (f)	[83]
**406**	(9′*Z*)-Lutein-5,6-epoxide	*C. officinalis* (f)	[84]
**407**	(*Z*)-Lutein dilaurate	*C. officinalis* (f)	[85]
**408**	(8′*R*)-Luteoxanthin	*C. officinalis* (f)	[83]
**409**	Mutatoxanthin	*C. officinalis* (f)	[83]
**410**	(9*Z*)-Neoxanthin	*C. officinalis* (f)	[83]
**411**	(13*Z*)-Neoxanthin	*C. officinalis* (f)	[84]
**412**	Neochrome	*C. officinalis* (f)	[83]
**413**	Phytoene	*C. officinalis* (f)	[85]
**414**	(*Z*)-Phytofluene	*C. officinalis* (f)	[85]
**415**	(all-*E*)-Phytofluene	*C. officinalis* (f)	[85]
**416**	(5′*Z*)-Rubixanthin	*C. officinalis* (f)	[84]
**417**	(5′*Z*, 9′*Z*)-Rubixanthin	*C. officinalis* (f)	[84]
**418**	Violaxanthin	*C. officinalis* (f)	[83]
**419**	Violaxanthin dipalmitate	*C. officinalis* (f)	[83]
**420**	Violaxanthin palmitate-stearate	*C. officinalis* (f)	[85]
**421**	(9*Z*)-Violaxanthin	*C. officinalis* (f)	[85]
**422**	(9*Z*)-Violaxanthin myristate	*C. officinalis* (f)	[85]
**423**	(all-*E*)-Violaxanthin	*C. officinalis* (f)	[85]
**424**	(all-*E*)-Violaxanthin myristate	*C. officinalis* (f)	[85]
**425**	(all-*E*)-Violaxanthin palmitate	*C. officinalis* (f)	[85]
**426**	(all-*E*)-Violaxanthin laurate	*C. officinalis* (f)	[85]
**427**	(all-*E*)-Violaxanthin dimyristate	*C. officinalis* (f)	[85]
**428**	(all-*E*)-Violaxanthin myristate-palmitate	*C. officinalis* (f)	[85]
**429**	Zeaxanthin	*C. officinalis* (f)	[83]
**430**	Zeaxanthin palmitate	*C. officinalis* (f)	[85]
**431**	Zeaxanthin myristate-palmitate	*C. officinalis* (f)	[85]
**432**	(all-*E*)-Zeaxanthin	*C. officinalis* (f)	[85]
**433**	(all-*E*)-Zeaxanthin myristate	*C. officinalis* (f)	[85]
**434**	(all-*E*)-Zeaxanthin dipalmitate	*C. officinalis* (f)	[85]
**435**	(all-*E*)-Zeinoxanthin	*C. officinalis* (f)	[85]
**436**	(all-*E*)-Zeinoxanthin myristate	*C. officinalis* (f)	[85]
**437**	Zeinoxanthin laurate	*C. officinalis* (f)	[85]
	Phenols		
**438**	*p*-Cymene	*C. arvensis* (ae) *C. officinalis* (ae)	[30,34]
**439**	*p*-Cymenene	*C. arvensis* (ae)	[34]
**440**	Carvacrol	*C. officinalis* (ae)	[30]
**441**	Thymol	*C. officinalis* (f)	[39]
**442**	*p*-Anethole	*C. officinalis* (f)	[35]
**443**	Estragole	*C. officinalis* (ae)	[36]
	Benzoic acids and derivatives		
**444**	2-Hydroxybenzoic acid (salycilic acid)	*C. officinalis* (f)	[87,88]
**445**	2-Methoxybenzoic acid (*o*-anisic acid)	*C. officinalis* (f)	[35]
**446**	4-Hydroxybenzoic acid (PHBA)	*C. officinalis* (f)	[87,88]
**447**	3,4-Dihydroxybenzoic acid (protocathechuic acid)	*C. officinalis* (f)	[87]
**448**	3-Methoxy-4-hydroxybenzoic acid (vanillic acid)	*C. officinalis* (f)	[87,88,89]
**449**	2,5-Dihydroxybenzoic acid (gentisic acid)	*C. officinalis* (f)	[87]
**450**	3,5-Dimethoxy-4-hydroxybenzoic acid (syringic acid)	*C. officinalis* (f)	[87,88,89]
**451**	1-*O*-*p*-Hydroxybenzoyl glucose	*C. officinalis* (l)	[90]
**452**	6-*O*-*p*-Hydroxybenzoyl glucose	*C. officinalis* (l)	[90]
**453**	1-*O*-Protocatechuoyl glucose	*C. officinalis* (l)	[90]
**454**	6-*O*-Protocatechuoyl glucose	*C. officinalis* (l)	[90]
**455**	1-*O*-Vanilloyl glucose	*C. officinalis* (l)	[90]
**456**	6-*O*-Vanilloyl glucose	*C. officinalis* (l,p)	[90,91]
	Hydroxycinnamates		
**457**	Cinnamic acid	*C. officinalis* (f)	[89]
**458**	2-Hydroxycinnamic acid (*o*-coumaric acid)	*C. officinalis* (f)	[89]
**459**	4-Hydroxycinnamic acid (*p*-coumaric acid)	*C. officinalis* (f) *C. tripterocarpa* (ae)	[87,88,89,92]
**460**	3,4-Dihydroxycinnamic acid (caffeic acid)	*C. arvensis* (ae) *C. officinalis* (f,l,p,s,r)	[75,87,88,89]
**461**	3-Methoxy-4-hydroxycinnamic acid (ferulic acid)	*C. officinalis* (f,p)	[87,89,91]
**462**	3-Hydroxy-4-methoxycinnamic acid (isoferulic acid)	*C. officinalis* (f,p)	[89,91]
**463**	3-*O*-*p*-Coumaroylquinic acid	*C. officinalis* (f)	[89]
**464**	1-*O*-Caffeoylquinic acid	*C. officinalis* (f)	[89]
**465**	3-*O*-Caffeoylquinic acid	*C. officinalis* (f,l,p,s,r)	[61,89,91]
**466**	4-*O*-Caffeoylquinic acid	*C. arvensis* (ae) *C. officinalis* (f,l,p,r) *C. suffruticosa* (ae)	[75,89,91]
**467**	5-*O*-Caffeoylquinic acid	*C. arvensis* (ae,r) *C. officinalis* (f,l,s,r) *C. suffruticosa* (ae)	[75,89,93]
**468**	1,3-Di-*O*-caffeoylquinic acid	*C. officinalis* (f,s,r) *C. suffruticosa* (ae)	[75,89]
**469**	1,5-Di-*O*-caffeoylquinic acid	*C. officinalis* (l) *C. suffruticosa* (ae)	[75,90]
**470**	3,4-Di-*O*-caffeoylquinic acid	*C. arvensis* (ae) *C. officinalis* (f) *C. suffruticosa* (ae)	[75,89]
**471**	3,5-Di-*O*-caffeoylquinic acid	*C. officinalis* (f,l,s,r)	[89]
**472**	4,5-Di-*O*-caffeoylquinic acid	*C. officinalis* (f,l,p,s,r)	[89,90,91]
**473**	1,3,5-Tri-*O*-caffeoylquinic acid	*C. officinalis* (f)	[89]
**474**	3,4,5-Tri-*O*-caffeoylquinic acid	*C. officinalis* (f)	[89]
**475**	5-*O*-Feruloylquinic acid	*C. arvensis* (ae,r) *C. officinalis* (f) *C. suffruticosa* (ae)	[75,89,93]
**476**	1,5-Di-*O*-feruloylquinic acid	*C. officinalis* (p)	[91]
**477**	1,5-Di-*O*-isoferuloylquinic acid	*C. officinalis* (p)	[91]
**478**	1-*O*-Caffeoyl glucose	*C. officinalis* (f)	[89]
	Coumarins		
**479**	Umbelliferone	*C. officinalis* (f,l)	[90,94]
**480**	Esculetin	*C. officinalis* (f,l)	[90,94]
**481**	Esculetin 6-*O*-^βD^Glc*p* (esculin)	*C. officinalis* (l)	[90]
**482**	Esculetin 7-*O*-^βD^Glc*p* (cichoriin)	*C. officinalis* (l)	[90]
**483**	Esculetin 7-*O*-(2″-*O*-^αL^Rha*p*)-^βD^Glc*p* (neoisobaisseoside)	*C. officinalis* (f)	[95]
**484**	Esculetin 7-*O*-(6″-*O*-^αL^Rha*p*)-^βD^Glc*p* (haploperoside A)	*C. officinalis* (f)	[95]
**485**	Scopoletin	*C. officinalis* (f,l) *C. tripterocarpa* (ae)	[90,92,94]
**486**	Scopoletin 7-*O*-^βD^Glc*p* (scopolin)	*C. officinalis* (f) *C. tripterocarpa* (ae)	[92,96]
**487**	Scopoletin 7-*O*-(2″-*O*-^αL^Rha*p*)-^βD^Glc*p* (haploperoside D)	*C. officinalis* (f)	[95]
**488**	Scopoletin 7-*O*-(6″-*O*-^αL^Rha*p*)-^βD^Glc*p* (isobaisseoside)	*C. officinalis* (f)	[95]
	Flavonols		
**489**	Kaempferol	*C. tripterocarpa* (ae)	[92]
**490**	Kaempferol 3-*O*-(6″-*O*-^αL^Rha*p*)-^βD^Glc*p* (nicotiflorin)	*C. arvensis* (ae,r)	[93]
**491**	Kaempferol 7-*O*-(6″-*O*-^αL^Rha*p*)-^βD^Glc*p*	*C. arvensis* (ae,r)	[93]
**492**	Quercetin	*C. officinalis* (f) *C. tripterocarpa* (ae)	[88,89,92,97]
**493**	Quercetin 3-*O*-^αL^Rha*p* (quercitrin)	*C. officinalis* (f,p)	[89,91]
**494**	Quercetin 3-*O*-^βD^Glc*p* (isoquercitrin)	*C. arvensis* (ae) *C. officinalis* (f,l,st)	[77,88,89,97,98]
**495**	Quercetin 3-*O*-(2″-*O*-Ac)-^βD^Glc*p*	*C. officinalis* (f)	[89]
**496**	Quercetin 3-*O*-(6″-*O*-Ac)-^βD^Glc*p*	*C. officinalis* (f,l,st)	[89]
**497**	Quercetin 3-*O*-(2″,6″-*O*-Ac_2_)-^βD^Glc*p*	*C. officinalis* (f)	[89]
**498**	Quercetin 3-*O*-^βD^Gal*p* (hyperoside)	*C. arvensis* (ae) *C. stellata* (w)	[72,77]
**499**	Quercetin 3-*O*-(2″-*O*-^αL^Rha*p*)-^αL^Rha*p*	*C. officinalis* (f,l)	[89]
**500**	Quercetin 3-*O*-(2″-*O*-^αL^Rha*p*)-^βD^Glc*p* (calendoflavobioside)	*C. arvensis* (ae) *C. officinalis* (f,p,l,st)	[75,89,91,97,98]
**501**	Quercetin 3-*O*-(3″-*O*-^αL^Rha*p*)-^βD^Glc*p* (calendoside II)	*C. officinalis* (f)	[99]
**502**	Quercetin 3-*O*-(4″-*O*-^αL^Rha*p*)-^βD^Glc*p* (calendoside I)	*C. officinalis* (f)	[99]
**503**	Quercetin 3-*O*-(6″-*O*-^αL^Rha*p*)-^βD^Glc*p* (rutin)	*C. arvensis* (ae,r) *C. officinalis* (f,st) *C. suffruticosa* (ae)	[75,89,91,93,97]
**504**	Quercetin 3-*O*-(2″,6″-*O*-^αL^Rha*p*_2_)-^βD^Glc*p* (manghaslin)	*C. officinalis* (f,p,st) *C. suffruticosa* (ae)	[75,89,91,98,100]
**505**	Isorhamnetin	*C. officinalis* (f)	[89,97]
**506**	Isorhamnetin 3-*O*-^αL^Rha*p*	*C. officinalis* (f)	[89,101]
**507**	Isorhamnetin 3-*O*-^βD^Glc*p*	*C. arvensis* (ae) *C. officinalis* (f,l,p,st)	[77,89,91,97,98]
**508**	Isorhamnetin 3-*O*-(2″-*O*-Ac)-^βD^Glc*p*	*C. officinalis* (f)	[89]
**509**	Isorhamnetin 3-*O*-(6″-*O*-Ac)-^βD^Glc*p*	*C. officinalis* (f,l,p,st)	[89,91]
**510**	Isorhamnetin 3-*O*-(2″,6″-*O*-Ac_2_)-^βD^Glc*p*	*C. officinalis* (f)	[89]
**511**	Isorhamnetin 3-*O*-(2″-*O*-^αL^Rha*p*)-^βD^Glc*p* (calendoflavoside)	*C. officinalis* (f,p,st)	[89,91,97,98]
**512**	Isorhamnetin 3-*O*-(3″-*O*-^αL^Rha*p*)-^βD^Glc*p* (calendoside IV)	*C. officinalis* (f)	[99]
**513**	Isorhamnetin 3-*O*-(4″-*O*-^αL^Rha*p*)-^βD^Glc*p* (calendoside III)	*C. officinalis* (f)	[99]
**514**	Isorhamnetin 3-*O*-(6″-*O*-^αL^Rha*p*)-^βD^Glc*p* (narcissin)	*C. officinalis* (f,p,st) *C. suffruticosa* (ae) *C. stellata* (w)	[72,75,89,91,97,98,101]
**515**	Isorhamnetin 3-*O*-(2″-*O*-^αL^Rha*p*)-^αL^Rha*p* (calendoflaside)	*C. officinalis* (f)	[97]
**516**	Isorhamnetin 3-*O*-(2″,6″-*O*-^αL^Rha*p*_2_)-^βD^Glc*p* (thyphaneoside)	*C. officinalis* (f,p,s,st)	[89,91,98,100,101]
	Anthocyanins		
**517**	Cyanidin 3-*O*-^βD^Glc*p*	*C. officinalis* (f)	[89]
**518**	Cyanidin 3,5-*O*-^βD^Glc*p*_2_	*C. officinalis* (f)	[89]
**519**	Cyanidin 3-*O*-(6″-*O*-^αL^Rha*p*)-^βD^Glc*p*	*C. officinalis* (f)	[89]
**520**	Delphinidin 3-*O*-^βD^Glc*p*	*C. officinalis* (f)	[89]
**521**	Malvidin 3-*O*-^βD^Glc*p*	*C. officinalis* (f)	[89]
**522**	Paeonidin 3-*O*-^βD^Glc*p*	*C. officinalis* (f)	[89]
**523**	Pelargonidin 3,5-*O*-^βD^Glc*p*_2_	*C. officinalis* (f)	[89]
**524**	Petunidin 3-*O*-^βD^Glc*p*	*C. officinalis* (f)	[89]
	Alkanes		
**525**	Tridecane	*C. officinalis* (ae)	[36]
**526**	Heptadecane	*C. arvensis* (ae) *C. officinalis* (f)	[33,39]
**527**	Octadecane	*C. officinalis* (f)	[39]
**528**	Nonadecane	*C. arvensis* (ae) *C. officinalis* (f)	[33,39]
**529**	Tricosane	*C. arvensis* (ae) *C. officinalis* (f,l)	[33,40]
**530**	Tetracosane	*C. arvensis* (ae) *C. officinalis* (f,l)	[33,40]
**531**	Pentacosane	*C. arvensis* (ae) *C. officinalis* (f,l)	[33,37,40]
**532**	Hexadecene	*C. officinalis* (ae,f,l)	[36,40]
**533**	Heptacosane	*C. officinalis* (f,l) *C. suffruticosa* (ae)	[40,41]
**534**	Octacosane	*C. officinalis* (f,l)	[40]
**535**	Nonacosane	*C. officinalis* (f,l) *C. suffruticosa* (ae)	[40,42]
**536**	Eicosane	*C. arvensis* (ae) *C. officinalis* (f)	[33,39]
**537**	Heneicosane	*C. arvensis* (ae)	[33]
**538**	Triacontane	*C. suffruticosa* (ae)	[42]
**539**	Untriacontane	*C. arvensis* (ae) *C. officinalis* (ae) *C. suffruticosa* (ae)	[41]
**540**	Tetratriacontane	*C. suffruticosa* (ae)	[42]
**550**	Cyclohexadecane	*C. officinalis* (f,l)	[40]
	Aliphatic alcohols		
**551**	(*Z*)-Hex-3-en-1-ol	*C. arvensis* (ae)	[34]
**552**	2-Methyl-6-heptene-3-ol	*C. officinalis* (f)	[29]
**553**	6-Methyl-5-heptene-2-ol	*C. officinalis* (f)	[35]
**554**	Hexadecan-1-ol	*C. suffruticosa* (ae)	[41]
**555**	9-Octadecen-1-ol	*C. suffruticosa* (ae)	[41]
**556**	6-Undecanol	*C. suffruticosa* (ae)	[41]
**557**	1-Tetracosanol	*C. suffruticosa* (ae)	[42]
**558**	1-Hexacosanol	*C. arvensis* (ae) *C. suffruticosa* (ae)	[41]
**559**	1-Octacosanol	*C. suffruticosa* (ae)	[41]
	Aliphatic Aldehydes and Ketones		
**560**	Nonanal	*C. arvensis* (ae) *C. officinalis* (ae,l)	[30,31,33]
**561**	Decanal	*C. arvensis* (ae)	[34]
**562**	(*E*, *E*)-2,4-Decadienal	*C. arvensis* (ae)	[34]
**563**	6-Methyl-5-heptene-2-one	*C. officinalis* (f)	[35]
**564**	2-Pentadecanone	*C. officinalis* (f)	[39]
**565**	(6*Z*,9*Z*)-Heptadeca-6,9-diene-5,11-dione	*C. officinalis* (f)	[102]
	Fatty Acids		
**566**	2-Methylpropanoic acid	*C. officinalis* (ae)	[36]
**567**	Capric acid	*C. officinalis* (f,l,s)	[103,104]
**568**	Lauric acid	*C. officinalis* (f,l,s)	[35,103,104,105]
**569**	Lauric acid methyl ester	*C. officinalis* (f)	[35]
**570**	Tridecanoic acid	*C. officinalis* (l)	[103,104]
**571**	Myristic acid	*C. officinalis* (f,l,s) *C. suffruticosa* (ae)	[41,103,104,105]
**572**	Myristic acid methyl ester	*C. officinalis* (f)	[35]
**573**	Myristic acid ethyl ester	*C. officinalis* (f)	[35]
**574**	Pentadecanoic acid	*C. officinalis* (f,l,s)	[103,104,105]
**575**	Palmitic acid	*C. arvensis* (ae) *C. officinalis* (f,l,s) *C. suffruticosa* (ae)	[39,41,103,104]
**576**	Palmitic acid methyl ester	*C. officinalis* (f)	[35]
**577**	Palmitic acid ethyl ester	*C. officinalis* (f)	[35]
**578**	*cis*-7-Hexadecanoic acid	*C. officinalis* (s)	[105]
**579**	Palmitoleic acid	*C. officinalis* (f,l,s)	[103,104,105]
**580**	Margaric acid	*C. officinalis* (f,l,s)	[103,104,105]
**581**	Margaric acid methyl ester	*C. officinalis* (f)	[35]
**582**	Stearic acid	*C. arvensis* (ae) *C. officinalis* (f,l,s) *C. suffruticosa* (ae)	[41,103,104,105]
**583**	Stearic acid methyl ester	*C. officinalis* (f)	[35]
**584**	Oleic acid	*C. officinalis* (f,l,s)	[103,104,105]
**585**	Elaidic acid	*C. officinalis* (s)	[105]
**586**	Linoelaidic acid	*C. officinalis* (f,l,s)	[103,104,105]
**587**	Linoleic acid	*C. arvensis* (ae) *C. officinalis* (s) *C. suffruticosa* (ae)	[41,105]
**588**	Linoleic acid methyl ester	*C. officinalis* (f)	[35]
**589**	Linolenic acid	*C. arvensis* (ae) *C. officinalis* (s) *C. suffruticosa* (ae)	[41,105]
**590**	Linolenic acid methyl ester	*C. officinalis* (f)	[35]
**591**	α-Calendic acid	*C. arvensis* (s) *C. officinalis* (f, l, s) *C. stellata* (f, s) *C. suffruticosa* (s) *C. tripterocarpum* (s) *C. maroccana* (s)	[16,103,104,105]
**592**	β-Calendic acid	*C. officinalis* (s)	[105]
**593**	9-Hydroxy-*trans*-10-*cis*-12-octadecadienic acid	*C. officinalis* (s)	[105]
**594**	Gondoic acid	*C. officinalis* (s)	[105]
**595**	Arachic acid	*C. officinalis* (f,l,s)	[103,104,105]
**596**	Heneicosanoic acid	*C. officinalis* (f,l)	[103,104]
**597**	Behenic acid	*C. officinalis* (f,l,s)	[103,104,105]
**598**	Tricosanoic acid	*C. officinalis* (f,l)	[103,104]
**599**	Tetracosanoic acid	*C. arvensis* (ae) *C. officinalis* (ae) *C. suffruticosa* (ae)	[41]
**600**	Lignoceric acid	*C. officinalis* (f,l)	[103,104]
**601**	Pentacosanoic acid	*C. officinalis* (f,l)	[103,104]
**602**	Octacosanoic acid	*C. suffruticosa* (ae)	[41]
	Chromanols		
**603**	2-Methyl-2-(4,8,12-trimethyltridecyl)chroman-6-ol (tocol)	*C. officinalis* (ae)	[106]
**604**	Tocol 5-methyl ester	*C. officinalis* (ae)	[106]
**605**	Tocol 7-methyl ester	*C. officinalis* (ae)	[106]
**606**	Tocol 8-methyl ester (δ-tocopherol)	*C. officinalis* (ae)	[106]
**607**	Tocol 5,7-dimethyl ester	*C. officinalis* (ae)	[106]
**608**	Tocol 5,8-dimethyl ester (β-tocopherol)	*C. officinalis* (ae)	[107]
**609**	Tocol 5,7-dimethyl ester (γ-tocopherol)	*C. officinalis* (ae)	[107]
**610**	Tocol 5,7,8-dimethyl ester (α-tocopherol)	*C. officinalis* (ae) *C. suffruticosa* (ae)	[42,107]
**611**	Plastoquinone	*C. officinalis* (ae)	[107]
**612**	Phylloquinone	*C. officinalis* (ae)	[107]
**613**	Ubiquinone	*C. officinalis* (ae)	[107]
	Organic Acids		
**614**	Malic acid	*C. arvensis* (ae,r)	[93]
**615**	Citric acid	*C. arvensis* (ae) *C. officinalis* (ae) *C. suffruticosa* (ae)	[41]
**616**	Quinic acid	*C. arvensis* (ae,r) *C. officinalis* (ae) *C. suffruticosa* (ae)	[41,93]
	Carbohydrates		
**617**	Threonic acid	*C. arvensis* (ae)	[41]
**618**	Ribose	*C. suffruticosa* (ae)	[41]
**619**	Tagatose	*C. arvensis* (ae) *C. officinalis* (ae) *C. suffruticosa* (ae)	[41]
**620**	Fructose	*C. arvensis* (ae) *C. officinalis* (ae) *C. suffruticosa* (ae)	[41]
**621**	Psicose	*C. arvensis* (ae) *C. officinalis* (ae) *C. suffruticosa* (ae)	[41]
**622**	Mannose	*C. arvensis* (ae) *C. officinalis* (ae) *C. suffruticosa* (ae)	[41]
**623**	Galactose	*C. arvensis* (ae) *C. officinalis* (ae) *C. suffruticosa* (ae)	[41]
**624**	Glucose	*C. arvensis* (ae) *C. officinalis* (ae) *C. suffruticosa* (ae)	[41]
**625**	Gluconic acid	*C. arvensis* (ae) *C. officinalis* (ae) *C. suffruticosa* (ae)	[41]
**626**	Galactaric acid	*C. arvensis* (ae) *C. officinalis* (ae) *C. suffruticosa* (ae)	[41]
**627**	Sucrose	*C. arvensis* (ae) *C. officinalis* (ae) *C. suffruticosa* (ae)	[41]
**628**	Cellobiose	*C. arvensis* (ae) *C. officinalis* (ae) *C. suffruticosa* (ae)	[41]
**629**	*scyllo*-Inositol	*C. officinalis* (ae) *C. suffruticosa* (ae)	[41]
**630**	*myo*-Inositol	*C. arvensis* (ae) *C. officinalis* (ae) *C. suffruticosa* (ae)	[41]
	Amino Acids		
**631**	Alanine	*C. officinalis* (f,l,st)	[108]
**632**	γ-Aminobutyric acid (GABA)	*C. arvensis* (ae) *C. officinalis* (ae) *C. suffruticosa* (ae)	[41]
**633**	Arginine	*C. officinalis* (f,l,st)	[108]
**634**	Aspartic acid	*C. officinalis* (f,l,st)	[108]
**635**	Asparagine	*C. officinalis* (f,l,st)	[108]
**636**	Histidine	*C. officinalis* (f,l,st)	[108]
**637**	Glutamic acid	*C. officinalis* (f,l,st)	[108]
**638**	Leucine	*C. officinalis* (f,l,st)	[108]
**639**	Lysine	*C. officinalis* (f,l,st)	[108]
**640**	Proline	*C. officinalis* (f,l,st)	[108]
**641**	Serine	*C. officinalis* (f,l,st)	[108]
**642**	Tyrosine	*C. officinalis* (f,l,st)	[108]
**643**	Threonine	*C. officinalis* (f,l,st)	[108]
**644**	Methionine	*C. officinalis* (f,l,st)	[108]
**645**	Phenylalanine	*C. officinalis* (f,l,st)	[108]
**646**	Valine	*C. officinalis* (f,l,st)	[108]
	Other Compounds		
**647**	3-Cyclohexene-1-ol	*C. officinalis* (ae)	[30]
**648**	3-Cyclohexene-1-ol 4-methyl ester	*C. officinalis* (l)	[31]
**649**	Loliolide	*C. officinalis* (f)	[109]
**650**	1,2,3,5,8,8α-Hexahydronaphthalene 6,7-dimethyl ester	*C. officinalis* (ae)	[36]
**651**	4-Methylacethophenone	*C. arvensis* (ae)	[34]
**652**	Tricyclene	*C. officinalis* (f)	[35]
**653**	1H-Benzocyclohepten-9-ol	*C. arvensis* (ae)	[41]
**654**	2-Pentyl furane	*C. arvensis* (ae)	[33]
**655**	1-Methyl ethyl hexadecanoate	*C. officinalis* (f,l)	[40]
**656**	Naphthalene	*C. suffruticosa* (ae)	[42]

^a^ Abbreviation used: Ac—acetyl; Ang—angeloyl; But—butyl; dCrt—dicrotaloyl; ^βD^Chi*p*—β-D-chinovopyranose; ^βD^Fuc*p*—β-D-fucopyranose; ^βD^Gal*p*—β-D-galactopyranose; ^βD^Glc*p*—β-D-glucopyranose; ^βD^GlcA*p*—β-D-glucuronopyranose; iBu—isobutyryl; iVal—isovaleroyl; ^βD^Glc*p*—β-D-glucopyranose; Mal—malonyl; Me—methyl; MBu—methylbutenoyl; MPe—methylpentenoyl; MPn—3-methyl-2-pentenoyl; MPr—methylpropanoyl; MSen—4-methylsenecioyl; ^αL^Rha*p*—α-L-rhamnopyranose; Sen—senecioyl; Tig—tigloyl. ^b^ Plant part: ae—aerial part, f—flowers, l—leaves, p—pollen, r—roots, s—seeds, st—stems.

**Table 3:** Source of polysaccharides of *C. officinalis*, extractant, monaccharide composition, yeld, molecular weight (MW), and fine structure.

Source, Extractant [Ref.]	Name	Ara	Gal	Glc	Man	Rha	Xyl	UA	Yield, %	MW, kDa	Fine Structure
Flowers, 0.5 M NaOH [119]	PS-I	34.2	41.0			24.8			0.08	15	rhamnoarabino-3,6-galactan
	PS-II	27.6	72.4						0.04	25	arabino-3,6-galactan
	PS-III	48.7	51.4						0.05	35	arabino-3,6-galactan
Flowers, water [120]	PSC-1	12.5	40.8	20.1		2.3		24.1	-	-	-
	PSC-2	11.0	35.2	14.0		0.9	1.6	37.2	-	-	-
	PSC-3	8.2	25.1	20.2		3.4		42.1	-	-	-
	PSC-4	7.5	35.7	11.5		3.1	1.0	40.5	-	-	-
	PSC-5	4.0	14.1	18.1		5.2		57.2	-	-	-
Industrial flowers waste, water [121]	-	7.5	6.1	1.7	1.8	0.5	1.2	58.3	8.90	40	-
Industrial flowers waste, 0.1 M HCl [122]	F	4.3	2.5		0.2	4.1	1.5	64.0	-	-	-
	HD	7.7	3.1		1.8	5.4	2.2	62.5	-	-	-

**Table 5:** Synopsis of known scientific information about metabolites of five *Calendula* species.

Group of Metabolites	*Calendula* Species ^a,b^				
	CA	CO	CSt	CSu	CT
Monoterpenes, sesquiterpenes, diterpenes as components of essential oils	AE	AE, F, L	F	AE	×
Sesquiterpene glycosides	AE	F	×	×	×
Triterpenes: sterols	AE	AE, F, L, R, S	×	AE	×
Triterpenes: ursanes and oleananes non-glycosidic	×	AE, F, L, R, S	×	AE	×
Triterpenes: glycosides	AE	AE, F, R	W	AE	×
Carotenoids	×	F	×	×	×
Benzoic acid derivatives	×	F, L, P	×	×	×
Hydroxycinnamates	AE	AE, F, L, P, R, S	×	AE	AE
Coumarins	×	F, L	×	×	AE
Flavonoids	AE, R	AE, F, L, P, R, S	W	AE	AE
Anthocyanins	×	F	×	×	×
Alkanes, aliphatic alcohols, aldehydes, ketones, fatty acids as components of lipophilic extracts	AE	AE, F, L, S	F	AE	×
Organic acids, carbohydrates	AE, R	AE	×	AE	×
Amino acids	AE	AE, F, L, St	×	AE	×
Chromanols	×	AE	×	AE	×
Polysaccharides	×	F	×	×	×

^a^ *Calendula* species: CA—*C. arvensis*; CO—*C. officinalis*; CSt—*C. stellata*; CSu—*C. suffruticosa*; CT—*C. tripterocarpum*. ^b^ Plant part: AE—aerial part; F—flowers; L—leaves; P—pollen; R—roots; S—seeds; St—stems; W—whole plant. Sign ‘×’ means no data found.

## Data Availability

Not applicable.

## Supplementary Materials

The following supporting information can be downloaded at: https://www.mdpi.com/article/10.3390/molecules27238626/s1, Table S1: Distribution of *Calendula* publications between research areas; Table S2: Top 10 cited articles aimed to *Calendula* research.
