# Supplementary material for: Marigold Metabolites: Diversity and Separation Methods of Calendula Genus Phytochemicals from 1891 to 2022
Source: Molecules. 2022 Dec 6;27(23):8626. doi: 10.3390/molecules27238626 (PMC9736270; doi:10.3390/molecules27238626)
Supplement: Supplementary file 1 [file molecules-27-08626-s001.zip › molecules-2014134-supplementary.pdf]

molecules

Supplementary Materials

Marigold Metabolites: Diversity and Separation Methods of *Calendula* Genus Phytochemicals from 1891 to 2022

Daniil N. Olennikov* and Nina I. Kashchenko

Table S1. Distribution of *Calendula* publications between research areas.

Research area	Publication percentage
Agricultural and Biological Sciences	37.8
Medicine	28.0
Pharmacology, Toxicology and Pharmaceutics	24.7
Biochemistry, Genetics and Molecular Biology	20.8
Chemistry	11.4

Table S2. Top 10 cited articles aimed to *Calendula* research.

First author, Title, Ref.	Citation count
Pommier, P. *et al*. Phase III randomized trial of *Calendula officinalis* compared with trolamine for the prevention of acute dermatitis during irradiation for breast cancer [2]	253
Akihisa, T. *et al*. Triterpene alcohols from the flowers of compositae and their anti- inflammatory effects [3]	241
Chaparzadeh, N. *et al*. Antioxidative responses of *Calendula officinalis* under salinity conditions [4]	217
Fronza, M. *et al*. Determination of the wound healing effect of *Calendula* extracts using the scratch assay with 3T3 fibroblasts [5]	197
Della Loggia, R. *et al*. The role of triterpenoids in the topical anti-inflammatory activity of *Calendula officinalis* flowers [6]	195
Ukiya, M. *et al*. Anti-inflammatory, anti-tumor-promoting, and cytotoxic activities of constituents of marigold (*Calendula officinalis*) flowers [7]	147
Wagner, H. *et al*. Immunstimulierend wirkende Polysaccharide (Heteroglykane) aus hoheren Pflanzen [8]	132
Yoshikawa, M. *et al*. Hypoglycemic, gastric emptying inhibitory, and gastroprotective principles and new oleanane-type triterpene oligoglycosides, calendasaponins A, B, C, and D, from Egyptian *Calendula officinalis* [9]	128
Ćetković, G.S. *et al*. Antioxidant properties of marigold extracts [10]	123
Jiménez-Medina, E. *et al*. A new extract of the plant calendula officinalis produces a dual in vitro effect: Cytotoxic anti-tumor activity and lymphocyte activation [11]	108
